# Cyto-molecular characterization of rDNA and chromatin composition in the NOR-associated satellite in Chestnut (*Castanea* spp.)

**DOI:** 10.1038/s41598-023-45879-6

**Published:** 2024-01-15

**Authors:** Nurul Islam-Faridi, George L. Hodnett, Tetyana Zhebentyayeva, Laura L. Georgi, Paul H. Sisco, Frederick V. Hebard, C. Dana Nelson

**Affiliations:** 1 Forest Tree Molecular Cytogenetics Laboratory, Southern Institute of Forest Genetics, USDA Forest Service, Southern Research Station, Texas A&M University, College Station, TX 77843 USA; 2 Department of Soil and Crop Sciences, Texas A&M University, College Station, TX 77843 USA; 3 The Schatz Center for Tree Molecular Genetics, Department of Ecosystem Science and Management, The Pennsylvania State University, University Park, PA 16802 USA; 4 Department of Forestry and Natural Resources, University of Kentucky, Lexington, KY 40546 USA; 5 Meadowview Research Farms, The American Chestnut Foundation, 29010 Hawthorne Drive, Meadowview, VA 24361 USA; 6 The American Chestnut Foundation, 50 North Merrimon Ave., Suite 115, Asheville, NC 28804 USA; 7 USDA Forest Service, Southern Research Station, Forest Health Research and Education Center, Lexington, KY 40546 USA; 8 USDA Forest Service, Southern Institute of Forest Genetics, Harrison Experimental Forest, 23332 Success Road, Saucier, MS 39574 USA

**Keywords:** Cytogenetics, Genetics

## Abstract

The American chestnut (*Castanea dentata*, 2*n* = 2*x* = 24), once known as the “King of the Appalachian Forest”, was decimated by chestnut blight during the first half of the twentieth century by an invasive fungus (*Cryphonectria parasitica*). The Chinese chestnut (*C. mollissima,* 2*n* = 2*x* = 24), in contrast to American chestnut, is resistant to this blight. Efforts are being made to transfer this resistance to American chestnut through backcross breeding and genetic engineering. Both chestnut genomes have been genetically mapped and recently sequenced to facilitate gene discovery efforts aimed at assisting molecular breeding and genetic engineering. To complement and extend this genomic work, we analyzed the distribution and organization of their ribosomal DNAs (35S and 5S rDNA), and the chromatin composition of the nucleolus organizing region (NOR)-associated satellites. Using fluorescent in situ hybridization (FISH), we have identified two 35S (one major and one minor) and one 5S rDNA sites. The major 35S rDNA sites are terminal and sub-terminal in American and Chinese chestnuts, respectively, originating at the end of the short arm of the chromosome, extending through the secondary constriction and into the satellites. An additional 5S locus was identified in certain Chinese chestnut accessions, and it was linked distally to the major 35S site. The NOR-associated satellite in Chinese chestnut was found to comprise a proximal region packed with 35S rDNA and a distinct distal heterochromatic region. In contrast, the American chestnut satellite was relatively small and devoid of the distal heterochromatic region.

## Introduction

Chestnuts belong to the genus *Castanea*, which consists of seven diploid (2*n* = 2*x* = 24) species native to temperate regions of the northern hemisphere^1,2^. The American chestnut [*Castanea dentata* (Marsh.) Borkh.], once known as the “King of the Appalachian Forest”, grew on over 800,000 km^2^ in its native range in eastern North America until its decimation by chestnut blight [caused by *Cryphonectria parasitica* (Murr.) Burr.] in the first half of the twentieth century. At one-point, American chestnut produced up to 25% of the hardwood lumber volume in the northeastern area of the United States and up to 15% in the Appalachian Mountains^3–6^. Chestnuts (*Castanea* spp.) are one of the most useful trees in the world, providing reliable, high carbohydrate food sources, stunningly beautiful, rot-resistant wood, and high-quality tannins for leather production^5–7^.

Chestnut blight is considered the worst man-made disaster in the history of the world’s forest ecosystems and directly resulted in the passage of the Plant Quarantine Act enacted by the United States Government in 1912 to reduce the chances of such a catastrophic event happening again^8^. The blight fungus was accidentally introduced in the late nineteenth century when chestnut seedlings from Japan were imported for commercial purposes^9,10^. The disease was first identified at the Bronx Zoo, New York, in 1904^11,12^, and in a span of about 50 years, some four billion American chestnut trees were lost to blight^13,14^. In contrast to American chestnut, Chinese chestnut (*C. mollissima* Blume) co-evolved with the pathogen and is resistant to chestnut blight^15^. Soon after the devastating impact of chestnut blight was realized, scientists began hybridizing Asian chestnuts with American chestnut to develop a blight-resistant chestnut for replanting^16,17^. Over the past few decades and especially more recently, interspecies backcross breeding and genetic engineering have been implemented collaboratively by the American Chestnut Foundation (TACF), the State University of New York, College of Environmental Science and Forestry (SUNY-ESF)^17–19^, and various forest health researchers and partners, with the goal of restoring American chestnut to its native range^16^.

Genetic linkage and physical maps have been developed for Chinese chestnut^20–23^ to identify and locate disease resistance genes for blight and *Phytophthora* root rot. In addition, Chinese chestnut genomes have been sequenced and assembled^24–27^, as has the American chestnut genome^28^. Unlike the comprehensive genome analysis (mapping, karyotyping, and sequencing) conducted in some plant species [e.g., Arabidopsis^29,30^, rice^31^, sorghum^32^, maize^33^, tomato^34^, wheat^35^, less has been done to study the chestnut genome, especially with respect to cytogenomics (molecular cytological analysis). Cytogenomic research complements genetic mapping and genome sequencing by detecting structural rearrangements such as inversions and translocations, which are common features between related species and their hybrids^33,36–38^. This knowledge is important for inter-species hybrid breeding especially if the target gene(s) are located on rearranged chromosome(s).

Fluorescence in situ hybridization (FISH), a cytogenomic technique to visualize specific DNA sequences in cells, has played an important role in revealing the structural organization and evolution of genomes, including localizing markers and/or genes to specific chromosomes^31,32,39–45^. In plants, ribosomal DNAs (rDNA) consisting of 18S–5.8S–25S/26S rDNA (referred to as 35S rDNA) and 5S rDNA occupy specific regions on one or several chromosomes of a genome. The major 35S rDNA associated with the nucleolus is in the NOR (nucleolus organizing region) and is often visually apparent as a secondary constriction, the primary constriction being at the centromere. The regions distal to the secondary constrictions are termed satellites and they can vary in size^46^. The rRNA genes within the 35S and 5S rDNA are arranged in tandem repeats of a few to several hundred or even thousands of copies^47^ and are often used as unique cytogenomics landmarks in FISH for karyotype analysis and studies of genome organization. In addition, their copy number variation and cytological positions on chromosomes provide evolutionary insight into relationships among species and the speciation process^36,48–53^.

To complement and extend the available genome information for American and Chinese chestnuts, we conducted a detailed cytogenomic characterization of the rDNA loci and their associated satellites in these species as well as their interspecific hybrid. Our specific objectives were as follows:

(i) to determine and compare the distribution and organization of the ribosomal gene loci (35S and 5S rDNAs) in American and Chinese chestnuts, and
(ii) to characterize and compare the structural phenotype, and chromatin composition of their NOR (35S rDNA)-associated satellites*.*

## Results

### Precis of results

**Figure 1:**
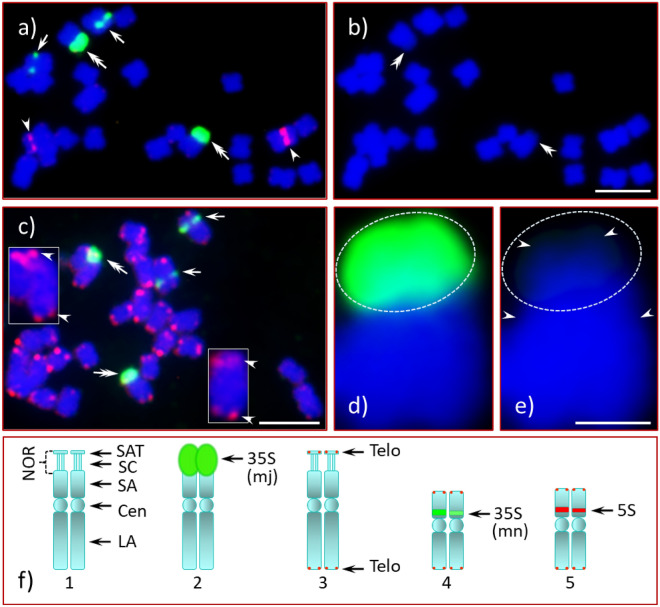
FISH with rDNA and telomere probes of American chestnut metaphase chromosomes. American Chestnut chromosome spread hybridized with 35S (green signals), 5S rDNA (red signals) and ATRS (telomere, red signals) probes, counter-stained with DAPI; (**a**) the mn-35S (arrows) and the 5S (arrowheads) sites, but not the mj-35S site (double arrows), have different signal intensities on the two homologues of their chromosomes; (**b**) DAPI stained chromosomes, the satellite and the NOR (double arrowheads) stained less intensely with DAPI; (**c**) telomere signals (red) observed on the terminal ends of every chromosome, enlarged image of the mj-35S rDNA (green signal, double arrows) bearing chromosomes with telomere signals (arrowheads) shown in inserts (top-left and bottom-slightly right); (**d**,**e**) enlarged image of one of the mj-35S bearing chromosomes with 35S signals [green, encircled, (**d**)]; (**e**) arrowheads (inside the white dotted circle) point at satellite, and primary constriction (centromere, just below the white dotted circle); (**f**) diagrammatic illustration of the rDNA bearing chromosomes including FISH signals, (**f1**) ideogram of the major NOR bearing chromosome pair, *SAT* satellite, *NOR* nucleolus organizing region, *SC* secondary constriction, *SA* short arm, *Cen* centromere, *LA* long arm, (**f2**) the same chromosome pair as in ‘**f1**’ with mj-35S signals, (**f3**) same with telomere signals, (**f4**) mn-35S rDNA chromosome pair with telomere signals, and (**f5**) 5S rDNA chromosome pair with telomere signals. Bars = 5 µm (**b**,**c**) and 1 µm (**e**). Note: These abbreviations are the same for each figure henceforth.

**Figure 2:**
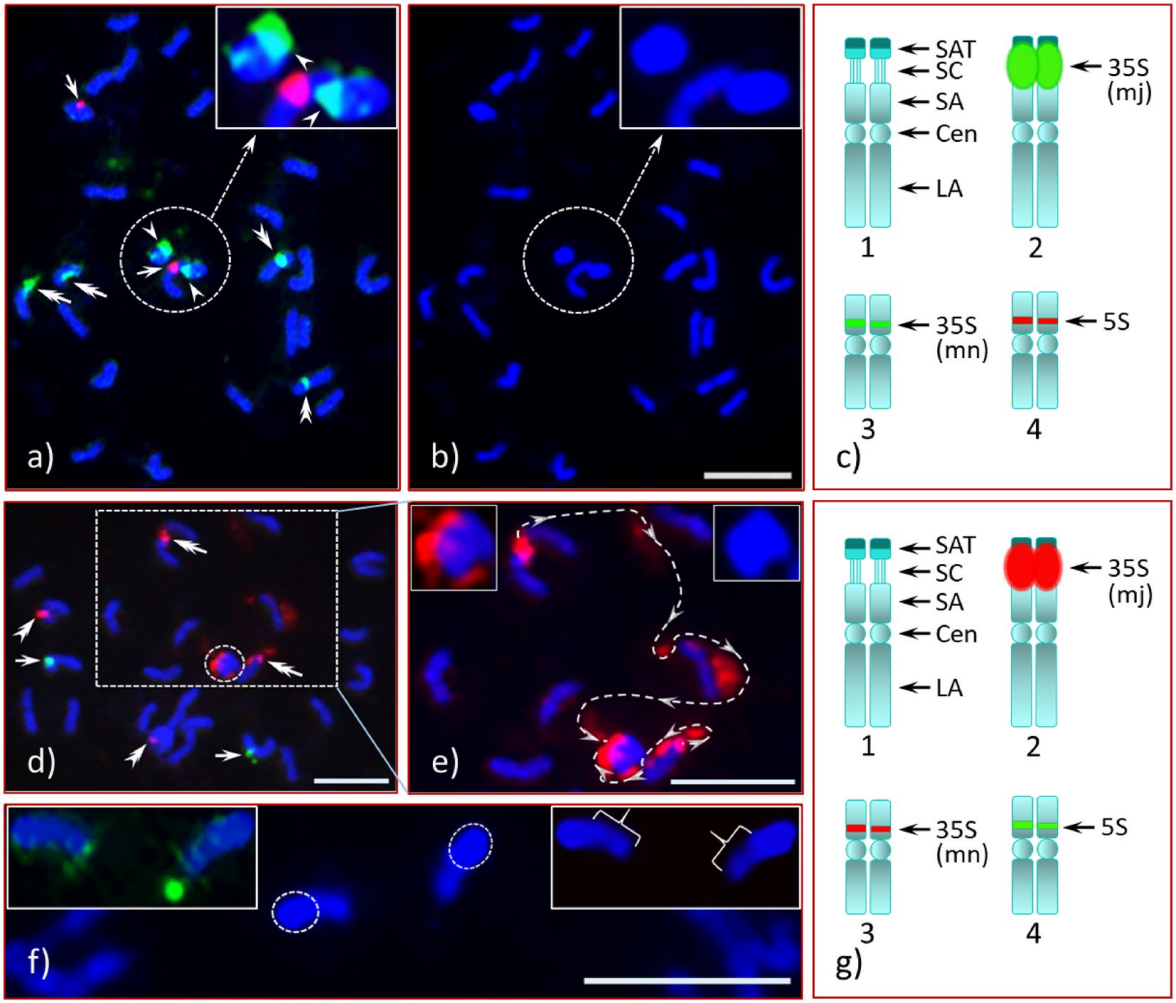
FISH of Chinese chestnuts (CC1 and CC2) with rDNA probes. Chinese chestnut trees, CC1 (**a**,**b**) and CC2 (**d**–**f**), chromosome spreads (pro-metaphase, mid-prophase) hybridized with 35S [green signals-CC1 (**a**) and CC2 (**f**) and red signals-CC2 (**d**,**e**)] and 5S [red signals-CC1 (**a**) and green-CC2 (**d**)] rDNA probes, counter-stained with DAPI; (**a**) mn-35S (double arrowheads) and 5S (arrows) sites exhibit different signal intensities, satellites with mj-35S (arrowheads) are encircled by white dotted lines [middle-center, top-right insert is an enlarged image of the same including one homologue with the 5S site (red signal)] and the double arrows pointing at the mother chromosomes; (**b**) DAPI image of the same cell as in ‘**a**’, insert (top-right) is an enlarged image of the satellites [white dotted circle (middle-center)]; (**d**) mj-35S rDNA signals associated with the satellites (encircled, middle-center) and the mother chromosomes (double arrows) are shown in rectangular box; (**e**) the image in the rectangular box ‘**d**’ was enlarged and further processed to enhance the red signals to show the path of the NOR (white dotted lines with arrowheads) of the mj-35S signals’ (red) connectivity of the mother chromosomes with the respective satellites, insert (top-left) is an enlarged image of the satellite pair with mj-35S signals, insert (top-right) is an enlarged DAPI image of the satellite pair; (**f**) a portion of an enlarged mid-prophase cell (DAPI image) of CC2 with two satellites (in the middle of the image), top-left insert is the same pair of satellites under green and blue filters showing scattered mj-35S signals (green) spread over the weakly stained region. This proximal region of the satellites stained weakly with DAPI and fluoresced less intensely under blue filter (top-right insert, braces). The heterochromatic region of the satellites (distal half, white dotted circles) stained bright blue with DAPI and fluoresced intensely under blue filter. Diagrammatic illustrations of the rDNA bearing chromosome pairs are given in ‘**c**’ (CC1) and ‘**g**’ (CC2). Abbreviations are same as in Fig. 1**f**. Bars = 5 µm (**b**,**d**,**e**) and = 1 µm (**f**).

**Figure 3:**
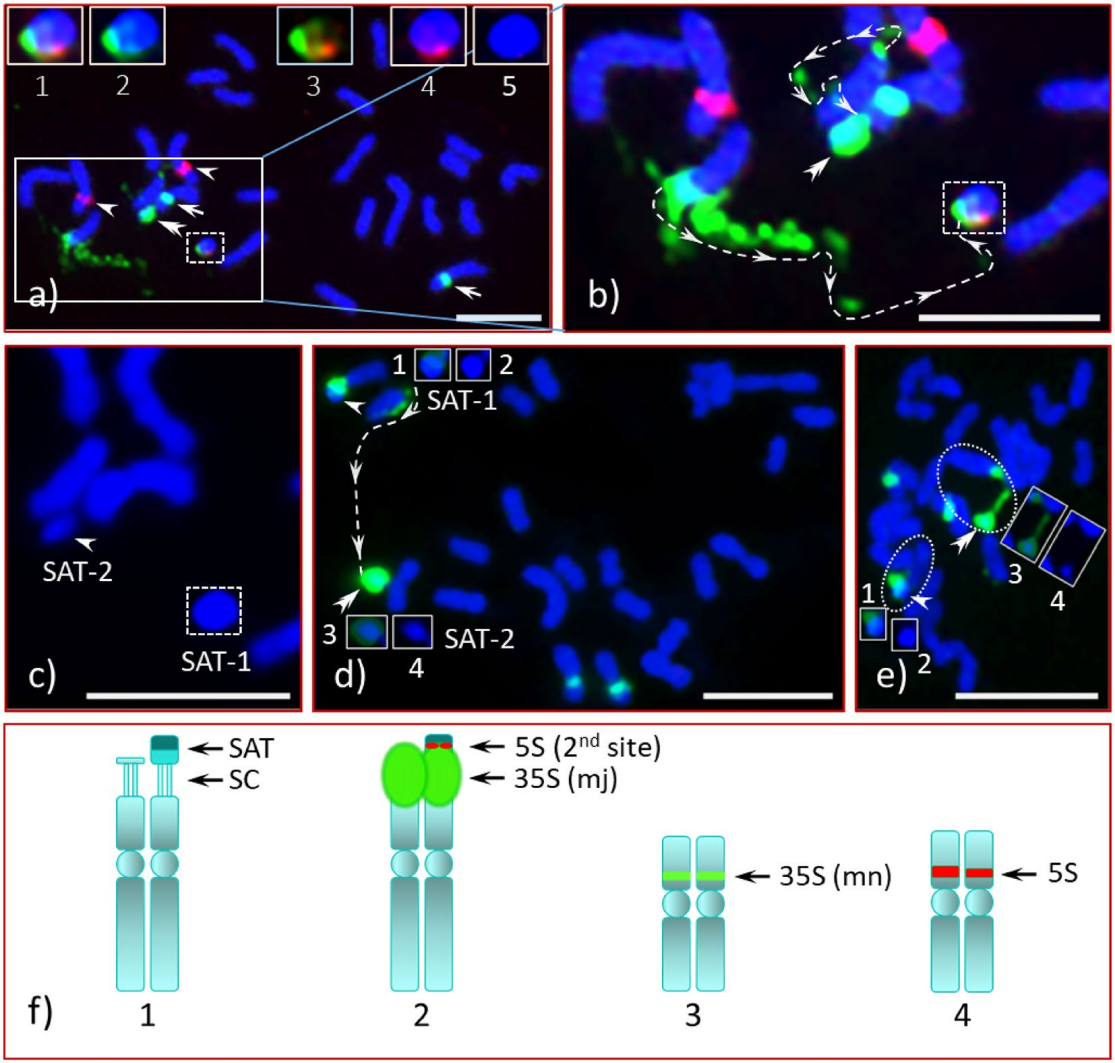
FISH of Chinese chestnut CC3 with rDNA probes. Pro-metaphase and early metaphase chromosome spreads of Chinese chestnut CC3 hybridized with 35S (green signals) and 5S (red signals) probes; a) two satellites [SAT-1 (white dotted lined box, inside the large rectangular box), SAT-2 (double arrowheads in the large rectangular box)] associated with the mj-35S signals, two mn-35S signals (arrows) and two 5S signals (arrowheads), an enlarged image of the SAT-1 under different filter shown in inserts (top, **1** to **5**) to demonstrate the 35S and the second 5S positions (localizations) in the satellite; image under red–green–blue (UV) filters (**a1**), green–blue filters (**a2**), red-green filters (**a3**), red-blue filters (**a4**) and blue filter (**a5**); (**b**) an enlarged image of the mj-35S bearing mother chromosomes and the satellites (large rectangular box in ‘**a**’) are seen to be connected (white dotted lines with arrowheads); (**c**) when FISH signals are removed (image process) leaving only the DAPI stain, both satellites can be seen clearly [an enlarged image of the satellites, SAT-1 (white dotted lined box) and SAT-2 (arrowhead)]; (**d**) an advanced pro-metaphase (or early metaphase) spread with 35S rDNA signals (green), SAT-1 chromosome is seen intact [arrowhead (top-left), SAT-1 with reduced green signal (**d1**) and DAPI image (**d2**)], SAT-2 is covered by green signal (double arrowhead), which is seen to be connected with its mother chromosome [(white dotted lines with arrowheads), SAT-2 with reduced green signal (**d3**) and DAPI image (**d4**)]; (**e**) an early metaphase, both SAT-1 (arrowhead) and SAT-2 (double arrowhead) seen to be clearly attached with respective mother chromosomes (each encircled with white dotted lines); SAT-1 with reduced green signal (**e1**, image processed further), DAPI image of SAT-1 (**e2**), SAT-2 with reduced green signal (**e3**, image processed further) and DAPI image (**e4**); (**f**) diagrammatic illustrations of the rDNA bearing chromosomes (abbreviations are same as in Fig. 1**f**). Bars = 5 µm.

**Figure 4:**
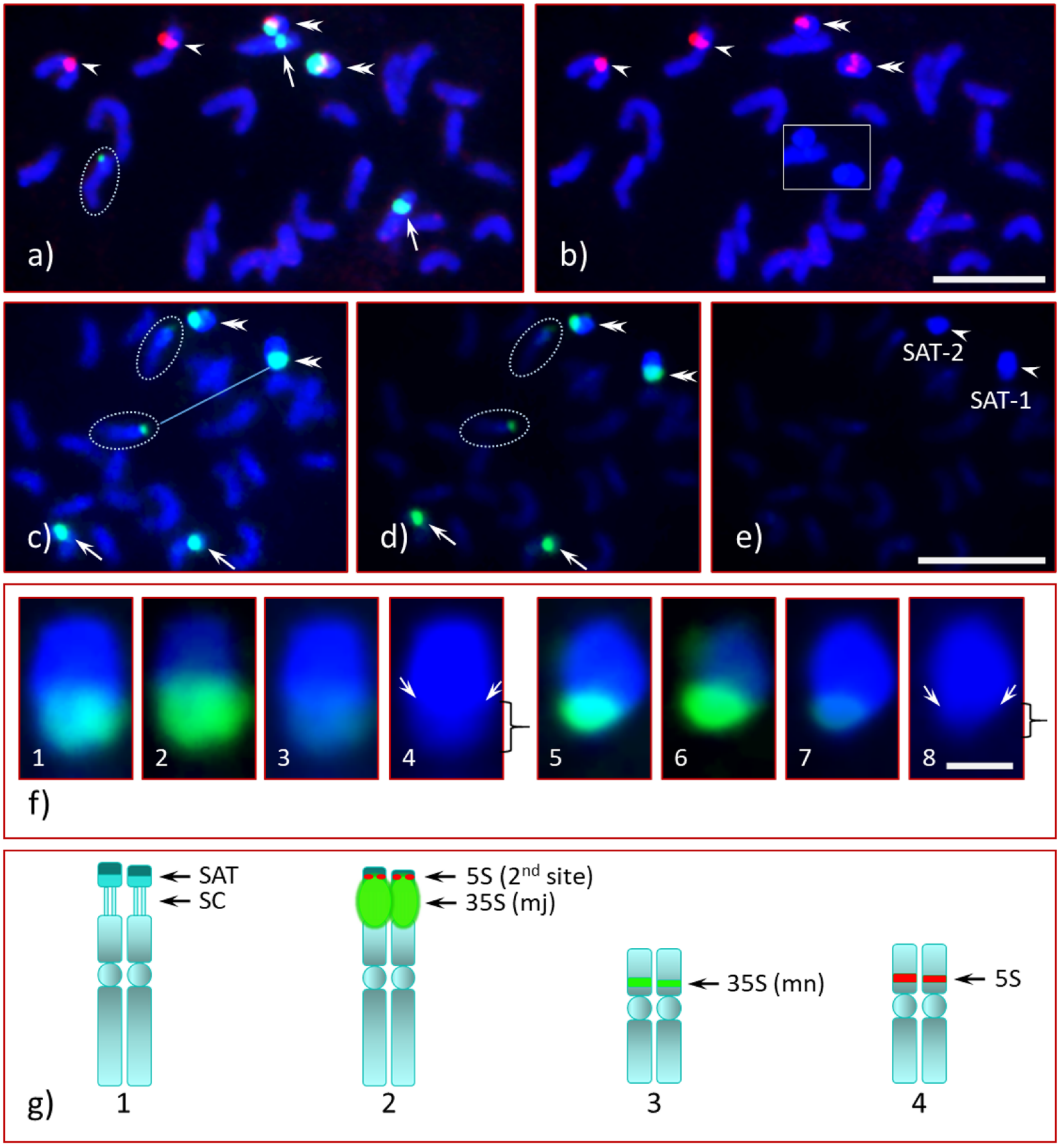
FISH of Chinese chestnut CC4 with rDNA probes. Chinese chestnut CC4 pro-metaphase chromosome spread hybridized with 35S (green) and 5S (red) rDNA probes; (**a**) SAT-1 and SAT-2 are different sizes (double arrowheads) with proximal 35S co-localized with the second 5S rDNA site (red), mn-35S site (arrows) and 5S site (arrowheads), one of the mother chromosomes encircled with white dotted line (middle-left); (**b**) same cell as in ‘**a**’ under red and blue filters shows two pairs of 5S sites (arrows and double arrowheads), the satellites with the second 5S site (double arrowheads) appear detached from the mother chromosomes, DAPI stained image of the satellites shown in insert (middle-slight right); (**c**) another cell of an early pro-metaphase spread with 35S signals. The satellites are marked by double arrowheads and the mother chromosomes are encircled (white dotted lines), the mn-35S site marked by arrows; (**d**) same cell as in ‘**c**’ with reduced DAPI (image processed further) to clearly demonstrate the 35S FISH signals, which are seen only in the weakly stained region of the satellites (brace, see enlarged DAPI image in ‘**f4**’ and ‘**f8**’, and clear separation between the two regions marked by bent arrows); (**e**) same cell as in ‘**c**' with reduced DAPI that clearly demonstrated the distal region of the satellites (arrowheads) is highly heterochromatic as it fluoresced intensely under blue filter; (**f**) enlarged images of SAT-1 and SAT-2 under different filters clearly demonstrating the proximal region of each satellite may be euchromatic as this region is integrated with the 35S rRNA gene (35S green signals), SAT-1 under green and blue filters (**f1**), image with reduced DAPI (**f2**), image with reduced green (**f3**) and DAPI image (**f4**) clearly shows differential DAPI intensities, the proximal region stained weakly (brace) and distal region stained intensely (highly heterochromatic), arrows path shows clear separation line between these two chromatin regions, for **f5**–**f8** (SAT-2) the description is same as in **f1**–**f4**, respectively; (**g**) diagrammatic illustration of the rDNA bearing chromosomes (abbreviations are same as in Fig. 1**f**). Bars = 5 µm (**b**,**c**), and = 0.5 µm.

**Figure 5:**
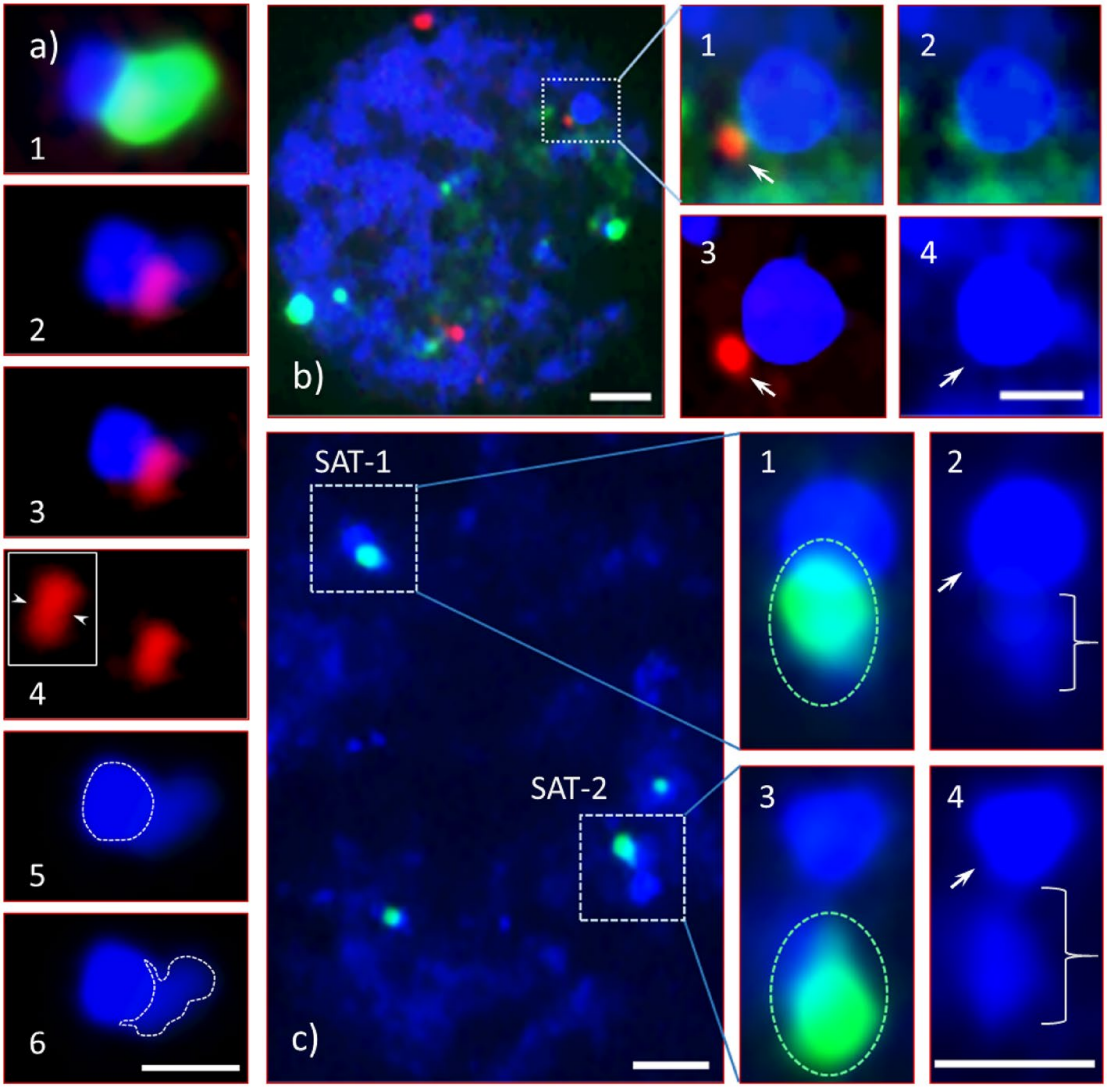
Chromatin (eu- and hetero-) composition in the NOR-associated satellite of Chinese chestnut. Weak (less intense) DAPI stained DNA of Chinese chestnut satellite associated with mj-35S and the second 5S rDNA in mid-prophase chromosome of CC3 (**a**), interphase nuclei of CC3 (**b**), and CC4 (**c**). Images in panel ‘**a**’ near side view, a mid-prophase SAT-1 of CC3 with the mj-35S (green) and the second 5S (red) rDNA signals. (**a1**) The green 35S signal obscures the red 5S (the second site) signal, but when removed through image process the 5S site is clearly visible (**a2**,**a3**,**a4**), and is associated with the region of the satellite which is visible as weak-blue DAPI stain just to the right of the second 5S site in ‘**a2'**; (**a3**) the less intense blue DAPI stain has been removed (through image processing) from ‘**a2**’; the 5S signal is seen adjacent (or on the periphery) of the heterochromatic region of the satellite i.e., the 5S signal is assumed to not be associated with the heterochromatin; (**a4**) image under red-filter, the 5S signal (arrows in insert-left show the separation point of two individual signals, one on each chromatid); (**a5**) heterochromatic region is encircled by white dotted line, and (**a6**) the less intense blue DAPI stain (may be euchromatic) of the proximal region is encircled in white dotted line. (**b**) An interphase nucleus of CC3 with 35S (green) and 5S (red) signals in highly decondensed DNA bodies; (**b1**–**b4**) enlarged and enhanced (to boost up the signals) image of the satellite (box in ‘**b**’) under different filters; (**b1**) superimposed image of red–green–blue (rgb) filters, the 5S signal (red, arrow) and dispersed green signals (35S rDNA) near the highly heterochromatic satellite (stained strongly with DAPI) as it fluoresced intensely under blue filter; (**b2**) the 5S rDNA signal has been removed (through image processing) leaving the dispersed 35S green signal; (**b3**) when the 35S rDNA and euchromatin signals have been removed from the image, the 5S signal (arrow) seen as a separate entity from the heterochromatic region of the satellite; (**b4**) image under blue filter, the heterochromatic region of SAT-1 (arrow) and scattered light blue euchromatic DNA. (**c**) A partial interphase nucleus of CC4 with its satellites SAT-1 and SAT-2 (boxed) with a mj-35S rDNA signal (green) in euchromatic DNA (stained weakly with DAPI, and fluoresced less weakly under blue filter); (**c1**,**c3**) enlarged images of SAT-1 and SAT-2, respectively, with a strong mj-35S signal (green, circled) with clear side view orientation of SAT-2 (**c3**) the mj-35S rDNA is clearly in the weak DAPI stained region that may be euchromatic; (**c2**,**c4**) the weak DAPI region (braces) of the respective satellites is observed below the heterochromatic region (arrows). Bars = 1 µm (**a6**,**b4**,**c4**) and = 2 µm (**b**,**c**).

**Figure 6:**
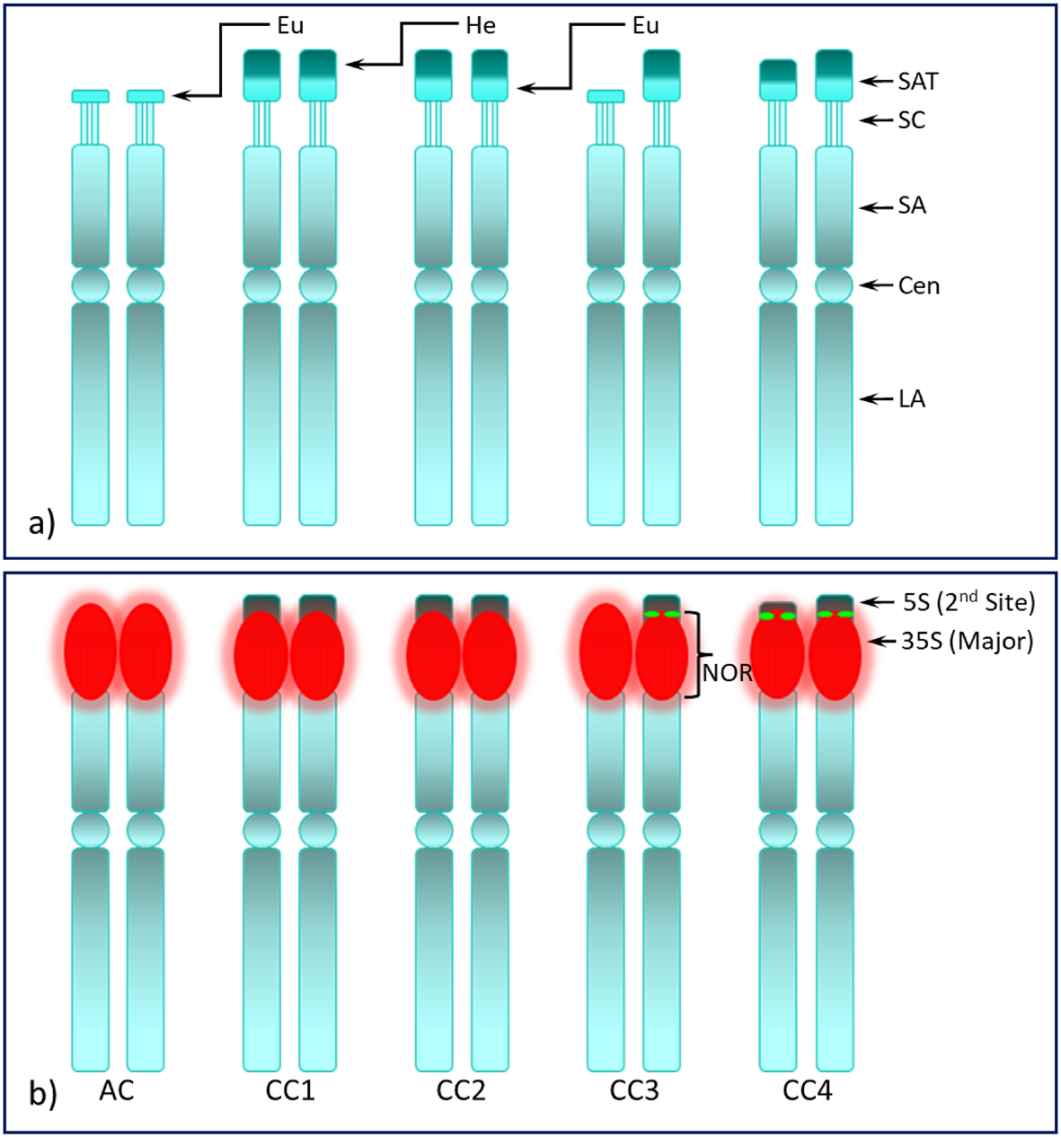
Structural illustration of the mj-35S rDNA bearing chromosomes of American and Chinese chestnuts. Structural illustration of the mj-35S rDNA homologues of American chestnut (AC) and four unrelated Chinese chestnut seedlings, open-pollinated progeny of parents ‘Veselicky’ (CC1), ‘Don Surrette’ (CC2), ‘AU-Cropper’ (CC3) and ‘Qing’ (CC4); (**a**) structural morphology of the major NOR (nucleolus organizer region), which is the site of the mj-35S rDNA locus (red signals in ‘**b**'), a second 5S site (green signal) co-localized distally with the mj-35S rDNA in one homologue with large satellite in CC3 and in both homologues in CC4; *SAT* satellite, *SC* secondary constriction, *SA* short arm, *Cen* centromere, *LA* long arm, *NOR* nucleolus organizing region, *Eu* euchromatin, *He* heterochromatin.

**Figure 7:**
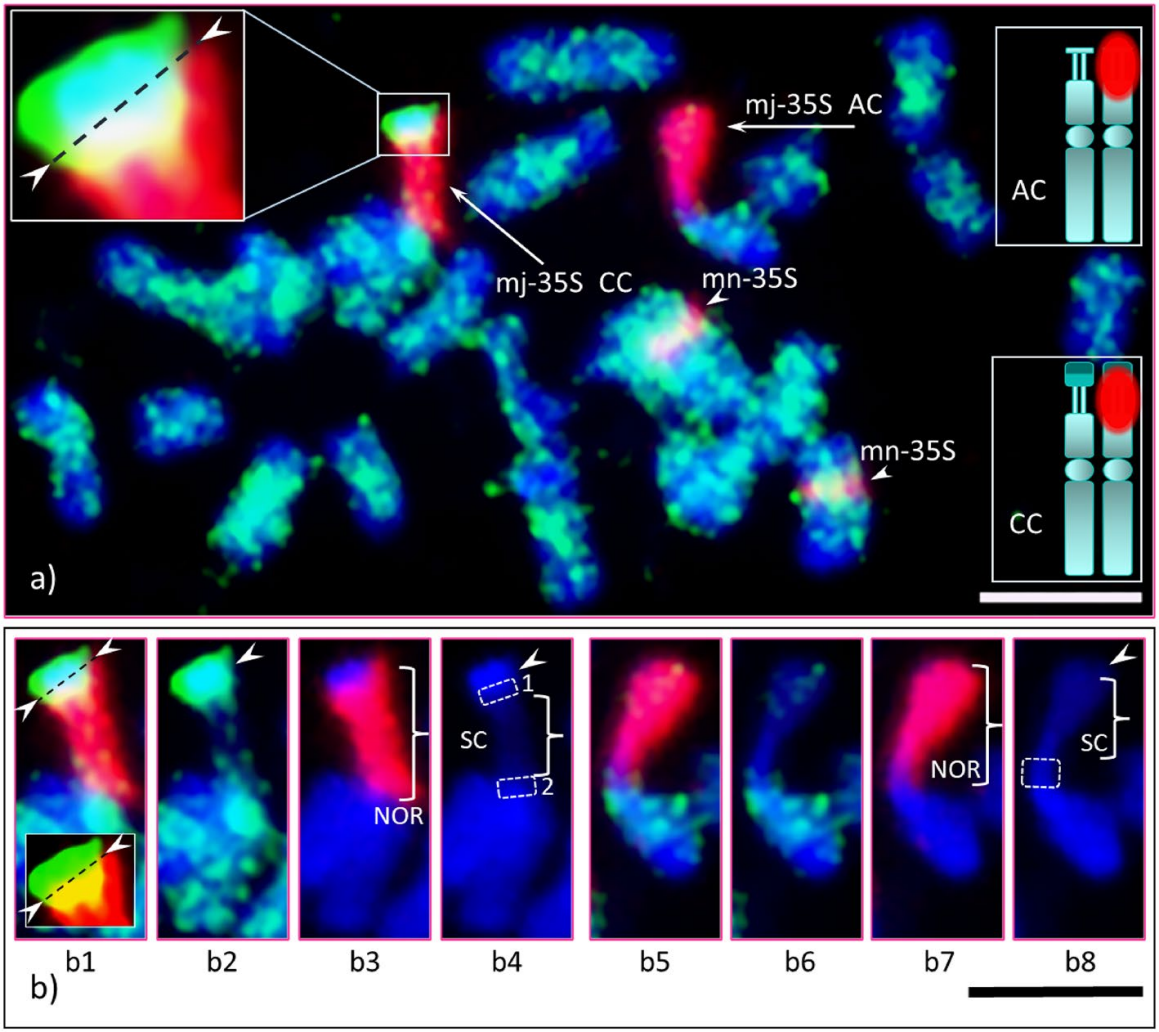
GISH of American and Chinese chestnuts F_1_ hybrid. Early metaphase chromosome spread of an F_1_ (Chinese chestnut × American chestnut) GISH-ed with Chinese chestnut total genomic DNA as probe and American chestnut as blocking DNA along with the 35S rDNA probe to study structural and organizational differences between the species in a common background; (**a**) scattered green signals (Chinese chestnut DNA) show across all 24 chromosomes; the mj-35S rDNA bearing chromosome of American chestnut marked as ‘AC’ (diagrammatic sketch shown in top-right insert), and the mj-35S rDNA bearing chromosome of Chinese chestnut marked as ‘CC’ (diagrammatic sketch shown in bottom-right inset). An enlarged image of the Chinese chestnut satellite shown in upper-left insert; a complete genomic hybridization (green signal) can be seen clearly in the heterochromatic region (distal half) and the proximal half, which hybridized with the 35S rDNA are separated by a dotted line (marked by arrowheads); further the DAPI stained heterochromatic region and the red signal from the 35S rDNA blended together through the green signal as diffused blue-greenish and yellowish signals, respectively. The mn-35S rDNA bearing chromosomes marked by arrowheads. (**b**) The individual mj-35S rDNA bearing chromosome of each species with NOR (marked by brace, **b3** and **b7**, respectively) under different filters: (**b1–4**) (Chinese chestnut chromosome; **b1**–**b4** are images of red–green–blue, green–blue, red–blue filters, and blue filter, respectively; (**b5–b8**) (American chestnut chromosome, same set of filters applied as for Chinese chestnut). (**b4**) The proximal region of the Chinese chestnut satellite and the distal region of the short arm shown in dotted boxes 1 and 2, respectively; the secondary constriction (SC, brace) which does not seem to stain with DAPI (i.e., DAPI negative). (**b8**) The distal part of the short arm of American chestnut shown in a dotted box, and its secondary constriction (SC, brace) also appears not to be stained with DAPI. Chinese chestnut satellite under red-green filters (without blue filter, i.e., DAPI) is given in the lower-bottom insert in ‘**b1**’. Bar = 5 µm.

One American chestnut (AC) and four Chinese chestnut accessions (CC1, CC2, CC3 and CC4) were analyzed for the occurrence of rDNA by FISH. Chromosome spreads of enzymatically digested root tips, mostly free of cell walls, nuclear membranes, and cytoplasmic debris, with well-separated chromosomes, provided good conditions for FISH^54,55^. The chromosomal morphology of all accessions was similar. Each accession has metacentric (m), near-metacentric (nm) and sub-metacentric (sm) chromosomes, and a NOR-associated satellite pair (see Supplementary Fig. S1A). Images for the chromosome spreads and an accompanying illustration of the morphology of the chromosomes containing rDNA loci for each accession are shown in Fig. 1 for AC and in Figs. 2, 3, 4, 5 for CC1 to CC4, respectively. Figure 6 presents a diagrammatic illustration of the major 35S rDNA bearing chromosome of AC and the North American accessions (NAAs) of Chinese chestnut, and the second 5S rDNA site in two NAAs (CC3 and CC4). Figure 7 shows GISH (genomic in situ hybridization) results for the F_1_ hybrid. Based on physical appearance we observed two different sizes of the satellites in CC3 and CC4, referred hereafter as SAT-1 for the larger of the pair and SAT-2 for the smaller (see Supplementary Fig. S1B). Additional FISH images and diagrammatic illustrations are presented in Supplementary Figures S2 to S7. A comprehensive summary is available in Supplementary information 2 (PowerPoint slides), 3 (PowerPoint slides' narratives), and 4 (PowerPoint audio-video presentation). Throughout the results and discussion, the 35S rDNA and 5S rDNA will be referred as 35S and 5S, respectively. In addition, the major 35S rDNA as mj-35S and the minor 35S rDNA as mn-35S.

### Ribosomal DNA in American and Chinese chestnuts

In the American chestnut (AC) two 35S sites (one major site and one minor site) and one 5S site (Fig. 1) were independently located on three different chromosomes. The mj-35S locus was located terminally with the FISH signal covering the end of the short arm of its chromosome, the secondary constriction (SC), and the entire satellite (Fig. 1a, double arrows). The mn-35S (Fig. 1a, green signals, arrows) and the 5S (Fig. 1a, red signals, arrowheads) sites were located proximally on the short arms. The signal intensity for the mj-35S appeared monomorphic on both homologues. In contrast, the signal intensities of the mn-35S and the 5S were dimorphic, that is, more intense for one homologue than for the other.

In Chinese chestnut CC1, we observed the same number of 35S and 5S loci (Fig. 2a) as in AC. Nevertheless, compared to AC the CC1 satellite was larger [Fig. 2a (encircled), an enlarged image (processed further) shown in upper-right corner], with its proximal half covered by the mj-35S green signal and distal half stained intensely with DAPI. In addition, these satellites appeared to be completely detached from their mother chromosomes (Fig. 2a, double arrows, middle-left). The mn-35S (Fig. 2a, double arrowheads) and the 5S (Fig. 2a, arrows) loci were observed in the same positions as in AC (compare Figs. 1a and 2a). All three of the rDNA loci appeared dimorphic in signal intensity.

Our result in CC1, namely the mj-35S locus mapping distal to the centromere (i.e., sub-terminal position), differs from a previous analysis of Chinese chestnut^56^, where the mj-35S locus was in a pericentromeric position. Given this observed difference, we analyzed three additional unrelated NAAs of Chinese chestnut, CC2, CC3 and CC4, and compared them to CC1 and AC. Interestingly, the four NAAs of Chinese chestnut (NAAs CC) did not differ in the position of the mj-35S locus but did vary in satellite length (see Supplementary Fig. S1B) and DNA composition. For additional details, see the animated and automated PowerPoint presentation, its accompanying narratives and the PPT audio-video in Supplementary information 2, 3 and 4, respectively. On one homologue of CC3 the mj-35S signal completely covered the SC and the satellite as in AC (Fig. 3a,b,d,e, double arrowheads, and Supplementary Figs. S4 and S5). The signal intensity of the mj-35S rDNA probes, which is monomorphic for the AC homologues (Fig. 1a,c, green signals, double arrows), is dimorphic for all Chinese chestnuts studied as illustrated in Fig. 2a (CC1), Fig. 2d (encircled middle-center, CC2), and 2e (enlarged image insert top-left, CC2), Fig. 3a,b,d,e (CC3) and Fig. 4a,c (CC4). Chromosomal positions for the mn-35S and the 5S sites for all NAAs of CC were similar as AC, including being dimorphic in their signal intensities. An additional 5S rDNA site in CC3 and CC4, located on the NOR-associated satellite, was observed linked distally to the mj-35S rDNA. Chinese chestnut accession CC3 was heteromorphic for this second 5S rDNA site as a single homologue exhibited the FISH signal [Fig. 3a1,a3,a4, (red signal, inserts top), and Fig. 5a2–a4,b (box, top-right), Fig. 5b1,b3 (red signal, arrow)] while signals were detected on both homologues of CC4 [Fig. 4a,b (red signals, double arrowheads)].

The chromosome spreads of American chestnut (AC) were highly condensed metaphase cells (Fig. 1) with a highly condensed NOR and associated satellite. The Chinese chestnut spreads (CC1, CC2, CC3 and CC4) were in pro- and early-metaphase stages with moderately condensed NORs. An intact stretched NOR can be clearly seen in early metaphase spreads [Fig. 3e (CC3) and Fig. 7 (F_1_ hybrid)]. As mentioned, the satellites of CC1 and CC4 do not appear to be associated with their mother chromosomes as there is little visual evidence they are connected. In addition, there is no 35S rDNA signal extending from the mother chromosomes of CC1 (Fig. 2a, double arrows, middle-left) to the satellites (encircled, middle of the image). However, several scattered 35S signals are visible in CC2 (Fig. 2d, red) when the image is enhanced, showing the path of the NOR that connects the respective mother chromosome to its satellite (Fig. 2e, white dotted lines along with arrowheads). This connection is clearly apparent in a slightly more condensed chromosome spread of CC3 where the mj-35S signal beginning on the short arm of the chromosome, passes through the SC and then extends into the proximal region of the SAT-1 (Fig. 3b, box), and completely covers SAT-2 (Fig. 3b, double arrowhead). Furthermore, when the spread quality was good, both chromatids at the NOR could be detected in mid- or late-prophase (or early pro-metaphase) as shown in Supplementary Fig. S5 and Supplementary information 2 and 4 (slide No. 3). The differences between Chinese and American chestnut revealed by FISH of mj-35S rDNA are summarized in a diagrammatic illustration in Fig. 6.

### Satellites of American and Chinese chestnuts

The satellites of American chestnut and all studied NAAs of Chinese chestnut (CC1, CC2, CC3 and CC4) were unique in their physical size and chromatin composition. In AC, the mj-35S signals covered the NOR and the satellite (Fig. 1a, double arrows). An enlarged image indicates the signal extended from above the satellite into the short arm of the chromosome proximal to the NOR (Fig. 1d, and Supplementary Fig. S3a–d). When the 35S signal was removed through image analysis, a small satellite was visible as a less intense DAPI stained region [Fig. 1b (double arrowheads), Fig. 1e (arrowheads in white dotted circle), and Supplementary Figs. S2e1 (insert, bottom), S2e2 (insert, third right), S3f (arrowheads in white dotted circle and arrowheads in rectangular box)]. Since the satellite is completely covered by the mj-35S signal, there was a question as to whether the satellite was intact. To answer this question, we used the ATRS (i.e., telomere) probe in FISH in addition to the 35S probe. We observed a pair of telomere signals (red, Fig. 1c) at the end of each chromosome, including the ends of the satellites under a Cy3 filter (Fig. 1c, arrowheads, inserts). This confirmed the integrity of the chromosomes.

Unlike AC satellites, which appeared to be euchromatic, satellites of CC1, CC2, CC3 and CC4, except for one homologue of CC3, appear to be composed distally of heterochromatin and proximally of euchromatic DNA, which is apparent in Fig. 5a and illustrated in Fig. 6. This distinction, euchromatic vs. heterochromatic regions, is clearly observed in early- and mid-prophase cells, and at times in late-prophase or early pro-metaphase cells (CC4, Fig. 4f4,f8) with appropriate chromatin condensation and DAPI stain. The chromatin composition is clearly visible in Fig. 2f for CC2, where the less intense DAPI stain is indicated by braces in the upper right inset while the intense DAPI staining identifying heterochromatin is indicated by the circled region (the distal half of the satellite). The CC4 satellites differ in size (Fig. 4f; see Supplementary Fig. S1B), with one satellite being slightly larger (SAT-1) than the other (SAT-2). The CC3 satellites were unique with SAT-2 being similar to AC, small and nearly invisible unless the image was processed further (i.e., by enhancing the blue channel) [Fig. 3c, an enhanced DAPI image (arrowhead), Fig. 3d4 (insert), Fig. 3e4 (insert)], while SAT-1 was like the other Chinese chestnut NAAs [Fig. 3a5 (an enlarged DAPI image), Fig. 3c (dotted box), Fig. 3d2 (insert), Fig. 3e2 (insert)].

### Ribosomal RNA genes and the proximal region of the satellite

To locate the mj-35S and the second 5S rDNA more precisely, we completed additional analyses using interphase nuclei and a prophase cell of the NAAs of Chinese chestnut. The enlarged image of a mid-prophase cell of a CC3 satellite (SAT-1) is shown in Fig. 5a1–a6. The mj-35S signal was very intense, obscuring a large portion of the satellite (the proximal half), but when removed through image processing, the chromatin composition becomes more apparent. The distal half of the satellite, composed of AT-rich heterochromatin that fluoresced intensely with DAPI under a blue filter [Fig. 5a5 (encircled), arrow in b4, c2, c4]. In contrast, the proximal half, which fluoresced less intensely [Fig. 5a6 (white dotted outlined), Fig. 5c2 (brace), Fig. 5c4 (brace)], might be euchromatic. The interphase nuclei of all NAAs of Chinese chestnut showed a characteristic spherical heterochromatic body [Fig. 5b, (SAT-1 of CC3, white dotted box), Fig. 5c (SAT-1 and SAT-2 of CC4, two white dotted boxes)], which is the distal portion of the satellite. The orientation of the mj-35S signals in CC4 (polar-side view, Fig. 5c1, SAT-1) obscured the spatial relationship of mj-35S rDNA and the satellite; however, the orientation of the SAT-2 (side view, Fig. 5c3) made it possible to determine unambiguously that the 35S rRNA genes were only in the weakly DAPI stained region of the satellite and clearly not in the heterochromatic region. Similarly, the second 5S rRNA locus in CC3 was definitely not associated with the heterochromatic region of SAT-1 [Fig. 5b3, also see Supplementary information 2 and 4 (slide No. 6)].

The mj-35S rDNA genic region was highly decondensed in interphase nuclei, as indicated by dispersed signals [Fig. 5b (green), and Supplementary Fig. S2c (red)], and are not associated with the heterochromatic region of the satellite (Fig. 5c4, arrow). The dispersed mj-35S signals along with or without the 5S rDNA locus coalesced as chromatin condensation progressed, revealing a visible connection of the mother chromosome to the satellite (Figs. 2e, 3b,d and Supplementary Figs. S2, S4 and S5).

### American × Chinese chestnut interspecific hybrid

In the interspecific F_1_ hybrid, chromosomes of American and Chinese chestnut could not be distinguished solely with GISH. Pericentromeric heterochromatic regions of some of the chromosomes were more densely covered by green hybridization signals (Fig. 7a), but it was not sufficient to determine the lineage of each chromosome (see review by Schubert et al.^57^). However, combining GISH with 35S rDNA FISH probes confirmed this cell was from an interspecific hybrid. This hybrid provided a unique opportunity to examine the compositional differences of the parental satellites under the same biological and cellular conditions. Consistent with previous observations, the mj-35S rDNA bearing chromosome of Chinese chestnut had a distinct portion of the satellite (bright green signal in GISH) extending beyond the 35S rDNA signal (Fig. 7a, mj-35S CC, red) that is heterochromatic, while the American chestnut’s satellite was completely covered by the signal (Fig. 7a, mj-35S AC). The DNA of each satellite was unique, as the genomic DNA of Chinese chestnut (GISH probe) hybridized to its satellite [bright green signal, Fig. 7a (boxes, top-left), Fig. 7b1,b2]; but a very limited hybridization occurred on the American chestnut satellite (Fig. 7b6, a few greenish dots of hybridization signals). Moreover, in the Chinese chestnut satellite, the distal region showed a strong DAPI stain, while the proximal region had a weaker stain and hybridized with the 35S rDNA. This indicates that the distal region is highly heterochromatic and noticeably distinct from the proximal region (as marked by dotted line in Fig. 7a, top-left box; and Fig. 7b1). Additionally, the middle segment of the NOR, which is the secondary constriction (SC), displayed no DAPI staining, meaning that it is DAPI negative (Fig. 7b2). This suggests that this region has a high GC content.

## Discussion

The number of rDNA loci (35S and 5S) and their organization with respect to copy numbers and chromosomal locations vary between and within species. This has led to their use for studying the chromosome structure and evolution of eukaryotic organisms^36,51,58–65^.

### Ribosomal DNA variation

We observed two 35S loci in both American and Chinese chestnuts, while the number of 5S loci varied. The American chestnut, as well as two NAAs of Chinese chestnut (CC1 and CC2) each had one 5S locus. Similar results have been reported in Japanese chestnut [*C. crenata* (Siebold & Zucc.)] and European chestnut [*C. sativa* (Mill.)]^56^. However, CC3 and CC4 had an additional 5S locus on the satellite linked distally to mj-35S. Gain or loss of a ribosomal locus is a common evolutionary feature in speciation^48,66^. The mj-35S site in AC was located at the terminal position, and those in the NAAs CC were located at the sub-terminal positions except for one homologue of CC3 located terminally. This homologue with terminal mj-35S may be pre-existent in the ancestral background of this accession’s maternal parent (i.e., ‘AU-Cropper’) or CC3 may represent a hybrid pollinated either by *C. crenata* or *C. sativa* since these two species were grown in the same orchard in Missouri with ‘AU-Cropper’. This would be consistent with Ribeiro et al.^56^, who found that both *C. crenata* and *C. sativa* carry a terminal mj-35S rDNA site. Interestingly, our results for the NNAs CC differ from that of Ribeiro et al.^56^, where the mj-35S and the second 5S loci in the European accession of *C. mollissima* were pericentromeric on the short arm. These authors suggested that these rDNA loci had been restructured during the evolution of *Castanea* species.

Our results agree with the FISH characterization of 11 *Quercus* species (members of the same family, Fagaceae) including *Q. robur*^59^, which was separated within the Fagales clade at about the same time (13.62 Mya) as *C. mollissima*^27^. An expressed sequence tag-consensus map between *Q. robur* and *C. sativa*^67^ further supports evolutionary relatedness between *Q. robur* and *Castanea* as does comparative mapping presented in Staton et al.^24^. Phylogenetically, *Quercus* and *Castanea* are closely related^68,69^, and molecular data suggested that *C. mollissima* was the first *Castanea* to evolve followed by *C. crenata*, *C. sativa*, and *C. dentata*^70^. *Castanea mollissima* may be a mosaic of land races derived from small founder populations in multiple domestication events combined with serial transfer of selections from one location to another, again with small founder populations. Under this scenario, the European accession of *C. mollissima*^56^ and the NAAs CC in our study would have presumably originated from different landraces. Based on this interpretation, it appears the European accession is likely to be a more recent derivative than the NAAs CC studied here and might have apparently resulted from a structural rearrangement of the mj-35S rDNA bearing chromosome through an inversion, translocation, or breakage-fusion event (for details see Supplementary information 1 and Supplementary Fig. S7).

In most eukaryotes the 35S (or 45S in animals) and the 5S rDNA loci are located independently of each other (known as S-type arrangement) and are rarely co-localized or linked (known as L-type association). In *C. mollissima,* we observed both L- and S-type associations. The L-type association was reported in only about 9% of 1791 plant species studied (see review by Garcia et al.^47^) and the 35S locus was predominantly distal to 5S, which is the reverse of what we found in accessions CC3 and CC4 where the 35S site was proximal to the 5S. Based on our FISH image analyses we suggest two possible scenarios of the L-type association of the 35S and 5S genes in chestnut. First, the distal portion of the 35S gene overlaps the proximal segment of the 5S gene (see Fig. 4a). Second, the 5S gene is embedded in the distal portion of the 35S gene [see Fig. 5a1, and compare with Fig. 5a2,a3; also see Supplementary information 2 and 4 (slide No. 6)]. Clearly, the organization of rRNA genes can be complex when both 35S and 5S rRNA genes are co-localized^53^. The organizational complexity of overlapping 35S and 5S rRNA genes can be demonstrated by extended DNA fiber FISH^71^ and/or molecular methods^72^. In addition, sequencing information might show the details of joint organization of these genes^47^.

### Ribosomal DNA signal variation

Homologous differences in rDNA signal intensities attributed to variation in rRNA gene copy number has been reported in several plant species^48,58,59^. In this study, while the 35S rDNA signal intensity for the mj-35S locus was about the same for both homologues of AC, it varied among the four NAAs of Chinese chestnut. Most striking was the large variation of signal intensity of the 35S rRNA gene between the homologues of CC3, suggesting dramatically different copy numbers. Dimorphic signal intensity in each of the mn-35S and 5S rDNA loci in AC and all four NAAs CC indicated copy number variation for these loci as well. Variation in copy numbers can result from unequal crossing over leading to higher copy numbers on one homologue relative to the other and may ultimately change the physical length of a locus, such as that observed for the NOR in other plant species [^64,73^; also observed in maize and ash, Islam-Faridi (unpublished)].

### Chromatin composition and structural organization of satellites

The DAPI stain intensity proportionally reflects the AT to GC enrichment of underlying genomic regions, where less intense DAPI staining (pale/weak blue) under blue/UV filter is associated with euchromatin and more intense DAPI staining (bright/strong blue) is associated with heterochromatin^34,74^. Variations in intensity of staining allow clear differentiation of chromatin composition in early stages of the cell cycle, such as interphase and occasionally in late prophase and early pro-metaphase. The proximal region of the NOR-associated satellites of CC2 (see Fig. 2f), CC3 (see Fig. 5a) and CC4 (see Fig. 4f) may be euchromatic since these regions stained less intensely with DAPI. This may also be true for CC1 because the 35S FISH signal also appears in the proximal region of the satellite (see Fig. 2a). In addition, the 35S rDNA probe hybridized in these regions as was demonstrated through image analysis enhancement. This hybridization revealed that the rRNA genes were present in the proximal region of the satellites and not in the heterochromatic region. As shown on Figs. 5a and 7, a clear demarcation boundary separates these regions [also see Supplementary information 2 and 3 (PPT file and narratives, respectively), and 4 (PPT audio-video), slide Nos. 5 and 6]. Moreover, the interphase nuclei of CC3 (see Fig. 5b) and CC4 (see Fig. 5c) explicitly showed that there was no association of rRNA genes with the distal heterochromatic DNA of the individual satellites.

The distal region of the Chinese chestnut NOR-associated satellite is heterochromatic as shown by the intense DAPI stain while the proximal region may be euchromatic as it stained less intensely. The AC satellite also exhibited a weak DAPI stain indicating it may be entirely euchromatic. This analysis was possible due to the increased resolution obtained in interphase and in the early stages of the mitotic cell cycle. Once the chromosomes reached their maximum or near-maximum condensation in metaphase (and sometimes in pro-metaphases), we could not differentiate the chromatin composition in the satellites. Garcia et al.^47^ reported that 2949 karyotypes of 1791 plant species had been developed based on metaphase FISH, and earlier Zoldoz et al.^59^ reported that the NOR-associated satellites were heterochromatic. It is unlikely FISH image analysis in these studies was carried out on the early stages of the cell cycle such that the details of the rDNA and chromatin organization could be determined. Additional research conducted in the early stages of mitosis of various plant species should shed light on whether the rRNA gene(s) resides in euchromatin of the NOR-associated satellites.

While the secondary constriction (SC) is traditionally viewed as the NOR site, using a 35S rDNA probe in CC we observed that the NOR spans the end of the short arm, the SC and the proximal portion of the satellite. For AC, the NOR occupies the end of the short arm, the SC and the entire satellite. A similar distribution of a mj-35S rDNA signal was observed in Baobab (*Adansonia digitata L.*)^55^ and ash species (*Fraxinus* spp.)^53^. Further, we observed that the SC did not stain with DAPI (i.e., DAPI negative) (see Fig. 7b4,b8). Three consecutive A-T base pairs are required to bind with DAPI^75^. This suggests that such arrangements are absent in this region. It has been reported that the mj-35S sites showed no stain with DAPI, but stained brightly with CMA3, indicating they are GC-rich^48,59^. In fact, it is the secondary constriction that is DAPI negative, not the complete 35S site. Most FISH data were based primarily on condensed metaphases, so the structural details of the NOR were missing.

We observed no differences in the satellites associated with the NOR between the homologues in CC1 and CC2, but the homologues differed in CC3 and CC4 (see Supplementary Fig. S1B). Because of natural outcrossing and inter-species hybridization in *Castanea*, the pollen parents of the CC3 and CC4 NAA seedlings might be a species other than *C. mollissima*. However, for CC1 and CC2 the pollen parents were most likely to be *C. mollissima* since the homologous pair of each satellite showed similar cytological features. For CC3, one homologue’s satellite was composed of only a small body of chromatin like AC that stained less intensely with DAPI, suggesting that this satellite entirely lost its heterochromatic portion. This homologue might have been pre-existent in the ancestral background of CC3, or it could be contributed either by *C. crenata* or *C. sativa* as discussed earlier. For CC4, the heterochromatic region of one homologue’s satellite was smaller than the other (see Fig. 4e,f; Supplementary Fig. S1Bc1–3), which suggests the smaller satellite (SAT-2) lost a noticeable portion of heterochromatic DNA. The SAT-2 bearing chromosome might have been contributed by *C. pumila*, (chinquapin, a sister clade to the chestnuts), as they are common in the area where CC4 was derived and are known to hybridize with chestnuts^76^. Additional research is needed to confirm this hypothesis. For example, if the smaller satellite of CC4 was consistent with that found in *C. pumila* then our findings of chromatin composition in the NOR-associated satellite would be supported. Further, the SAT-1 of the NAAs CC that we studied were observed to be similar in physical size and chromatin composition. Regardless of the source of the NOR-associated satellite, the proximal region was stained less intensely with DAPI and hybridized with 35S rDNA composed of euchromatin. Additional research conducted in the early stages of mitosis should shed light on whether the rRNA gene(s) resides in the weakly stained chromatin of the NOR-associated satellites.

GISH of the F_1_ hybrid between Chinese and American chestnuts failed to differentiate the chromosomes of the two parental species. Cross-hybridization was observed in both genomes among all chromosomes (see Fig. 7a) showing the lack of significant differences in repetitive DNAs (see review by Schubert et al.^57^). Nevertheless, the presence of the distal segment of the large satellite extending beyond the 35S signal in the NOR bearing chromosome of Chinese chestnut enabled us to discriminate between Chinese and American chestnuts for this region of the genome. We observed the large satellite on the NOR bearing chromosomes in all four NAAs of Chinese chestnut was on just a single chromosome in the F_1_ hybrid. In addition, when Chinese chestnut DNA was used as a labeled probe, the satellite exhibited a strong bright green signal under a FITC filter (see Fig. 7b2) and intense blue signal under a DAPI filter (see Fig. 7b4, arrow). This suggests that this distal region is AT-rich and thus highly heterochromatic^34,74^. This area remains condensed throughout the cell-cycle, except when it replicates in late S-phase^77^. It is worthy to note that this region might be species and/or chromosome specific^39,78,79^. The molecular methods of cloning followed by FISH could confirm this notion. Heterochromatin is typically gene-poor and transcriptionally silent compared to euchromatin, and it was thought to serve as a graveyard for selfish mobile DNAs. However, recent studies suggest otherwise, i.e., heterochromatin contributes to important roles in cellular functions, such as transcriptions, chromosome segregation and long-range chromatin interactions (see reviews by Grewal and Jia^80^; Tamaru^81^). Currently, the biological significance of the heterochromatic DNA in the Chinese chestnut satellite is unclear. DNA sequence data for this satellite should begin to provide some information as to its significance and possible role in differentiating the species. In addition, a comparative study involving an AC SAT in CC cytoplasm and vise-versa should shed light on any interaction in a different genetic background.

## Summary and conclusions

We characterized the organization and distribution of the ribosomal RNA genes (35S rDNA and 5S rDNA), and the structural and chromatin composition of the NOR-associated satellites in American and Chinese chestnuts. Two 35S rDNA loci (one major and one minor) and one 5S rDNA locus were identified in each species. We also identified a second 5S rDNA site linked distally to the major 35S rDNA locus in two of the four unrelated North American accessions of Chinese chestnut. Based on prophase and pro-metaphase FISH, we demonstrate that in both species the minor 35S and 5S loci were located proximally near the centromeric region in two non-homologous, near-metacentric chromosomes. The major 35S rDNA was in terminal and sub-terminal positions in American and Chinese chestnuts, respectively. The satellite of Chinese chestnut may be determined to be composed of euchromatic and heterochromatic regions of DNA while the American chestnut satellite may be composed of euchromatin. Immunostaining of histone markers will validate the chromatin composition within the satellites. This endeavor will be integrated into our next phase of cytogenomic research. In Chinese chestnut, we found that the distal segment of the major 35S gene, with or without the second 5S, was exclusively located in the proximal region of the satellite. This is the first instance, to our knowledge, that a plant species’ satellite has been identified to have unique chromatic regions of DNA. To clearly demonstrate and summarize these findings, we have prepared a fully animated and automated PowerPoint presentation that includes narratives, and an audio-video component (see Supplementary information 2, 3 and 4, respectively).

Further research using rDNA in FISH in early stages of the cell cycle and image analysis in different plant species should shed light on the chromatin composition in the NOR-associated satellites, and whether the ribosomal gene integration with the euchromatin in the NOR-associated satellite is a common feature.

Since the American and Chinese chestnut genomes have been sequenced, the use of molecular cytology (e.g., oligo-FISH) should further broaden our understanding of the structure and organization of the individual chromosomes of the *Castanea* species. Oligo-FISH probes will be used in future research to continue the study of the structural organization of the genome. In addition, a comparative FISH study of the DNA composition of the species’ major 35S rDNA bearing chromosome using genetically mapped probes should reveal the genomic and genic differences that could be essential to optimize interspecies backcross breeding strategies for the incorporation of disease resistance into American chestnut.

## Materials and methods

### Plant materials

Open-pollinated seeds were collected from two Chinese chestnut trees (Veselicky, in Pittsburgh PA and Don Surrette, in Pisgah, NC), two Chinese chestnut cultivars (‘AU-Cropper’ and ‘Qing’, in an orchard at the University of Missouri, Columbia, MO), and one American chestnut tree (provided by Dr. Thomas Kubisiak, Southern Institute of Forest Genetics, Saucier, MS). Control-pollinated F_1_ hybrid seeds (Chinese × American) were provided by Mr. William White of The American Chestnut Foundation, Meadowview, VA. Seeds were germinated and grown in potting mix (MetroMix SunGrow SB-650, Sun Gro Horticulture, Agawam, MA, USA) in a greenhouse in College Station, TX. In this study we refer to these Chinese chestnut trees (parents and their progeny) as North American accessions (NAAs) of Chinese chestnut (or NAAs CC), and specifically Veselicky as CC1, Don Surrette as CC2, ‘AU-Cropper’ as CC3, and ‘Qing’ as CC4. The American chestnut and F_1_ hybrid progeny are referred to as AC and F_1_, respectively. All parental Chinese chestnut trees utilized in this research have been established in the United States for decades through public breeding programs of USDA-ARS Beltsville, MD, Connecticut Agricultural Experiment Station, University of Missouri or the American Chestnut Foundation. These trees and samples of their progeny have been used by publicly supported researchers over these decades and are considered part of the public domain. This research was conducted in full compliance with IUCN Policy Statement on Research Involving Species at Risk of Extinction and the Convention on the Trade in Endangered Species of Wild Fauna and Flora. For example, only non-lethal samples were utilized, no samples were collected in the wild, and no plants or samples were moved across international boundaries.

### Slide preparation for FISH

To accumulate metaphase chromosomes, actively growing root tips, about 1 cm long and with a milky white transparent appearance, were placed in the dark in an aqueous solution saturated with 0.8% of α-bromonaphthalene for 2 h or a solution of 2.5 mM hydroxyquinoline for 3.5 h followed by fixation in 4:1 (95% ethanol:glacial acetic acid). Fixed root tips were enzymatically processed to prepare chromosome spreads using a modification of the procedure of Jewell and Islam-Faridi^82^. The enzyme solution consisted of 40% (v/v) Cellulase (C2730, Sigma-Aldrich, St. Louis, MO, USA), 20% (v/v) Pectinase (P2611, Sigma-Aldrich), 2% (w/v) Cellulase RS (SERVA Electrophoresis GmbH, Heidelberg, Germany), 3% (w/v) Cellulase R10 (Yakult Pharmaceutical, Ind. CO., Tokyo, Japan), 1% (w/v) Macerozyme (Yakult Pharmaceutical, Ind. CO., Tokyo, Japan), and 1.5% (w/v) Pectolyase Y23 (Kyowa Chemical Products, CO., Osaka, Japan) in a 0.01 M Citrate buffer (pH 4.5). Digested root tips were washed four times with the same buffer and then macerated on ethanol (95%) cleaned glass slides (1 root per slide) in a drop of 3:1 (ethanol:glacial acetic acid). Chromosome spreads of individual seedlings were prepared as described elsewhere^82^.

### Azure-B stain

The chromosome spreads were stained with 1% Azure-B (A4043, Sigma, St. Louis, MO) in 0.01 M Phosphate buffer pH (7.0) for 4 min at RT followed by a rinse in ddH_2_O. The slides were then dried overnight in a 37 C incubator. The preparation was made permanent with a drop of Permount (SP15, Fisher Scientific, Fair Lawn, NJ) under a glass cover-slip (22 × 40 mm). Chromosome spreads were viewed in bright field microscopy (AxioImager M2, Carl Zeiss, Göttingen, Germany) with a 63× plan apo-chromatic objective. Digital images were recorded under a green filter. The resolution of the captured images was increased to 300 dpi from 72, converted to black and white, and processed using Adobe Photoshop (Adobe Systems Inc., Broadway, NY).

### Fluorescence in situ hybridization

Three cloned DNAs were used as fluorescent in situ hybridization (FISH) probes: 18S–5.8S–26S rDNA of maize^83^, 5S rDNA of sugar beet including the spacer region^84^, and *Arabidopsis*-type telomere repeat sequence (TTTAGGG)n (ATRS) (kindly provided by Dr. T. McKnight, Department of Biology, Texas A&M University). The probes were labeled with biotin-16-dUTP (Biotin-Nick Translation Mix, Roche, Mannheim, Germany) and/or digoxigenin-11-dUTP (Dig-Nick-Translation Mix, Roche, Mannheim, Germany) using the manufacturer’s instructions. Agarose gel electrophoresis was used to check the fragment sizes of the probe DNA and labeled nucleotide incorporation was verified by dot-blotting. For genomic in situ hybridization (GISH), genomic DNA of the Chinese chestnut tree ‘HHR3T1’ was labeled with digoxigenin-11-dUTP as described elsewhere^85^.

Standard FISH or GISH techniques were utilized as reported previously^54,85^. Hybridization sites of biotin-labeled and digoxigenin-labeled probes were detected using Cy3-conjugated streptavidin (Jackson ImmunoResearch Laboratories, West Grove, PA, USA) and fluorescein-conjugated sheep anti-digoxigenin (Roche, Mannheim, Germany), respectively. Slides were counter-stained with DAPI (4 μg/ml) in McIlvaine buffer, pH 7.0 (9 mM citric acid, 80 mM Na_2_HPO_4_, 2.5 mM MgCl_2_), and a small drop (~ 10 μl) of Vectashield (Vector Laboratories, Burlingame CA, USA) was added to prevent photo-bleaching of the fluorochromes. Three FISH experiments and one GISH experiment were conducted as specified below.

FISH: Experiment 1: American chestnut (AC) and Chinese chestnut (CC1) chromosome spreads were probed with 35S and 5S rDNAs. Experiment 2: The ATRS probe was used along with the 35S rDNA (as a control) on the AC chromosome spreads to investigate the terminus of the major 35S rDNA bearing chromosome, since it was completely covered by 35S signal. Experiment 3: Three additional NAAs of Chinese chestnut (CC2, CC3 and CC4) were probed with 35S and 5S rDNAs.

GISH: Since the NOR-associated satellite was heterochromatic in the NAAs CC but not in AC, we used GISH of an F_1_ hybrid to provide a common background with 35S rDNA and DAPI staining to characterize the differences in the satellites’ structural organization and chromatin composition. Total genomic DNA from Chinese chestnut (DNA of the tree ‘HHR3T1’, a ramet of cv ‘Kuling’ provided by Dr. T. Kubisiak, Southern Institute of Forest Genetics, Saucier, MS) was used as a probe while total genomic DNA from American chestnut (DNA of the tree ‘GMBig’ also provided by Dr. T. Kubisiak) was used as blocking DNA to differentiate Chinese and American chestnut genomes in the F_1_ hybrid.

### Digital image capture and process

Fluorescent in situ hybridization images of chromosome spreads were viewed using a 63× plan apochromatic objective or a 100× plan-neofluor objective on an epi-fluorescence microscope (AxioImager M2, Carl Zeiss Inc., Göttingen, Germany) fitted with suitable filter sets (Chroma Technology Corp., Bellows Falls, VT, USA). Digital images were captured and recorded with a Cool Cube 1 high performance charge-coupled device (CCD) camera (MetaSystems Group Inc., Boston, MA, USA). Images were pre-processed with ISIS v5.1 (MetaSystems Group Inc., Boston, MA USA) and further processed with Adobe Photoshop CC2019 (Adobe Inc., San Jose, CA USA) after increasing the resolution from 72 to 300 dpi.

### Supplementary Information

Supplementary Information 1.

Supplementary Information 2.

Supplementary Information 3.

Supplementary Information 4.

## Data Availability

All data generated and analyzed for this study are included in this article as well as its Supplementary Document files.
