# Publisher Correction: Cyto-molecular characterization of rDNA and chromatin composition in the NOR-associated satellite in Chestnut (*Castanea* spp.)

**DOI:** 10.1038/s41598-024-62279-6

**Published:** 2024-05-27

**Authors:** Nurul Islam-Faridi, George L. Hodnett, Tetyana Zhebentyayeva, Laura L. Georgi, Paul H. Sisco, Frederick V. Hebard, C. Dana Nelson

**Affiliations:** 1 Forest Tree Molecular Cytogenetics Laboratory, Southern Institute of Forest Genetics, USDA Forest Service, Southern Research Station, Texas A&M University, College Station, TX 77843 USA; 2 Department of Soil and Crop Sciences, Texas A&M University, College Station, TX 77843 USA; 3 The Schatz Center for Tree Molecular Genetics, Department of Ecosystem Science and Management, The Pennsylvania State University, University Park, PA 16802 USA; 4 Department of Forestry and Natural Resources, University of Kentucky, Lexington, KY 40546 USA; 5 Meadowview Research Farms, The American Chestnut Foundation, 29010 Hawthorne Drive, Meadowview, VA 24361 USA; 6 The American Chestnut Foundation, 50 North Merrimon Ave., Suite 115, Asheville, NC 28804 USA; 7 USDA Forest Service, Southern Research Station, Forest Health Research and Education Center, Lexington, KY 40546 USA; 8 USDA Forest Service, Southern Institute of Forest Genetics, Harrison Experimental Forest, 23332 Success Road, Saucier, MS 39574 USA

Correction to: *Scientific Reports* 10.1038/s41598-023-45879-6, published online 15 January 2024

The Supplementary Information files published with this Article contained errors. During Production, Supplementary Information file 2 was inadvertently replaced by Supplementary Information file 4. As a result, Supplementary file 2 was omitted. The omitted file now accompanies the original Article.

The original Article and accompanying Supplementary Information files have been corrected.

