# Supplementary material for: Cyto-molecular characterization of rDNA and chromatin composition in the NOR-associated satellite in Chestnut (Castanea spp.)
Source: Sci Rep. 2024 Jan 15;14:980. doi: 10.1038/s41598-023-45879-6 (PMC10789788; doi:10.1038/s41598-023-45879-6)
Supplement: Supplementary file 1 — Supplementary Information 1. [file 41598_2023_45879_MOESM1_ESM.docx]

**Supplementary information – 1**

Cyto-molecular characterization of rDNA and chromatin composition in the NOR-associated satellite in Chestnut (*Castanea* spp.)

Nurul Islam-Faridi^1^, George L Hodnett^2^, Tetyana Zhebentyayeva^3,4^, Laura L Georgi^5^, Paul H Sisco^6^, Frederick V Hebard^5^, C Dana Nelson^7,8^

^1^ Forest Tree Molecular Cytogenetics Laboratory, Southern Institute of Forest Genetics, USDA Forest Service, Southern Research Station, Texas A&M University, College Station, TX 77843, USA.

^2^ Department of Soil & Crop Sciences, Texas A&M University, College Station, TX 77843, USA.

^3^ The Schatz Center for Tree Molecular Genetics, Department of Ecosystem Science and Management, The Pennsylvania State University, University Park, PA 16802, USA.

^4^ Department of Forestry and Natural Resources, University of Kentucky, Lexington, KY 40546, USA.

^5^ Meadowview Research Farms, The American Chestnut Foundation, 29010 Hawthorne Drive, Meadowview, VA 24361, USA.

^6^ The American Chestnut Foundation, 50 North Merrimon Ave., Suite 115, Asheville, NC 28804, USA.

^7^ USDA Forest Service, Southern Research Station, Forest Health Research and Education Center, Lexington, KY 40546, USA.

^8^ USDA Forest Service, Southern Institute of Forest Genetics, Harrison Experimental Forest, 23332 Success Road, Saucier, MS 39574, USA.

**Corresponding author:** Nurul Islam-Faridi

E-mail: m.n.faridi@udsa.gov or nfaridi@tamu.edu; Phone: 979-862-3908

In this section, we lay out some additional results about the characterization of the major 35S rDNA and the NOR-associated satellites in American chestnut (AC) and four North American Accessions of Chinese chestnut (CC1, CC2, CC3 and CC4) including six hypotheses for how the chromosomal rearrangement may have occurred involving the mj-35S rDNA bearing chromosome in a European accession of Chinese chestnut [1].

**Additional Results and Discussion:** Chromatin condensation initiates when the cell enters the mitotic stage of the cell cycle beginning in prophase and continues through metaphase where chromosomes reach their most contracted state [2]. In plants, the 35S rRNA gene consists of 18S – 5.8S – 25S/26S sub-units. The subunits 18S and 5.8S are separated by ITS1 (internal transcribed spacer 1) while the ITS2 (internal transcribed spacer 2) separates 5.8S and 25S/26S subunits. Each unit of the 35S rRNA gene is separated by IGS (intergenic spacer) whose length is highly variable in species of the same genus and even in the individuals of the same species [3, 4]. The major 35S-associated NOR is the very last region of a chromosome to condense and it is fragile [5]. Depending on the condensation of the NOR, the arrangement of the mj-35S rRNA gene and its fragility may yield numerous FISH signals of varying sizes (large to small or to very small) which can be seen in different stages of the cell cycle from interphase to early or to late pro-metaphase, and sometimes in early metaphases [6, 7]. The mj-35S bearing chromosome cannot be identified until pro-metaphase (or sometimes in late-prophase) when the chromatin is sufficiently condensed and image analyzed (Adobe Photoshop) [Figs. 2e (dotted lines along with arrowheads), 3b (dotted lines along with arrowheads), main text; S2e (encircled with white dotted line), and S4a (encircled with white dotted line for the SAT-2 chromosome and white dotted line along with arrowheads for the SAT-1 chromosome)]. These signals become more organized in pro-metaphase [Figs. 3d, and 3e, (main text)] and appear as a slightly stretched single signal in late pro-metaphase or early-metaphase (Fig. 7, main text) reaching its maximum contraction at the end of metaphase when chromatids (also known as sister chromosomes) of each chromosome become distinguishable (Fig. 1, main text; Figure S3). A diagrammatic illustration of condensation of major 35S associated NOR (i.e., mj-35S rDNA site) from interphase to metaphase is given in Figure S6.

The American chestnut (AC) satellite is very small when compared to Chinese chestnut (CC1, CC2, CC3 and CC4) and is covered completely by the mj-35S signal [Figs. 1a (main text, green, double arrows), S2a, S2c, and S2e (red, double arrows; double arrowheads in interphase nucleus in S2c)]. Two relatively large circular balls of red signals (Cy3 fluorochrome), that cover the individual AC satellites, are visible throughout the cell cycle from interphase to metaphase (Fig. S2). This ball of red signal could be the reflection of accumulation of numerous copies of 35S rRNA genes aggregated around the satellite as illustrated in Figures S3e, and S3h. A tiny DAPI stained blue chromosomal body (i.e., satellite associated with the mj-35S) is visible when the red signals are removed through image analysis [Figs. S2e, (bottom box in panel 2Se1) and the 3rd box (to the right) in panel S2e2]. An enhanced DAPI image of the same cell is shown in Figure S2f (arrows, boxes).

A good FISH image can provide a detailed view of the structural composition of a chromosome (Fig. S5). Two strings of distinct 35S FISH signals at the NOR, each representing individual chromatids, are clearly visible in mid/late-prophase of CC3 (Fig. S5a, and compare with Figs. S5b, and S5c). An enlarged image of the SAT-2 (satellite associated with mj-35S) bearing chromosome of CC3 is shown in Figures S5d, and S5e [the original image is taken from a complete cell shown in Figure S4a (SAT-2 marked as double arrowheads)]. It clearly shows two lobes (marked by two bent arrows, Fig. S5d, compare with Figs. S5e, and S5f1) of round to oval shape of 35S FISH signals (green) that undoubtedly contain many copies of 35S rRNA genes accumulated at SAT-2, which is covered by the FISH signal. The light DAPI stained SAT-2 (euchromatic DNA only) becomes visible when the green FISH signals are removed through image analysis (Fig. S5f3). Further, in a quality chromosome spread in metaphase when cells are in maximum condensation, the chromatids of each chromosome can be distinguished. An enlarged image of one of the mj-35S bearing chromosomes of AC from Figure 1 (main text) is shown in Figure S3 to demonstrate further the details of the mj-35S signal, satellite, and chromatids. We observed two large lobes of oval green signals of the mj-35S site that were overlapping each other (Fig. S3). A longitudinal white dotted line is drawn between the chromatids, each with an individual lobe of signal at the satellite (Fig. S3b and S3d). The end of the short arm of the chromosome stained with DAPI blends through the green signal that loses the brightness of the green fluorochrome [Fig. S3b, (encircled with blue oval dotted line within the large oval circle)], but the uniformity of the signal intensity is clear throughout the signal area when DAPI is removed through image analysis (Fig. S3d). The uniformity of the signal intensity in each lobe further demonstrates that the 35S rRNA gene is distributed uniquely in the NOR of each chromatid. A diagrammatic illustration of each lobe of the 35S FISH signals (green spiral line drawing) of individual NOR of each chromatid is given in Figure S3e (encircled with white dotted line).

The primary constriction (centromeric position) is apparent in the upper-middle of the chromosome [Figs. S3c, (a slight curvy white dotted line marked with arrowheads), and S3f, (arrowheads, just below the oval-shaped white dotted line)]. The end of the short arm (the satellite and the telomere end, marked by arrowheads in oval-shaped white dotted line in Fig. S3f) is clearly seen in the enhanced DAPI image in the rectangular box (Fig. S3f, arrowheads). Diagrammatic illustrations of this mj-35S rDNA bearing chromosome of AC are shown in Figure 3g (SAT = satellite, NOR = nucleolus organizing region, SA = short arm, CEN = centromere, LA = long arm) and Figure 3h [35S signals are shown two balls of wool (like two lobes, spiral lines of green)].

**Structural Rearrangement:** As mentioned earlier, comparing our results with Ribeiro et al. [1], some structural rearrangements must have taken place to reposition the mj-35S rDNA and the second 5S rDNA loci during divergence of Chinese chestnut. Structural rearrangement is a common feature during evolution, producing new variants and species [8 – 10]. Here we suggest six alternative hypotheses for how this rearrangement might have occurred (Figs. S7a-g).

**Hypothesis-I:** A complete whole arm pericentromeric inversion (hypothesis Ia, Fig. S7a), either from a prototype of the Chinese chestnut accessions from North America (e.g., CC3 or CC4) or a complete inversion followed by a reciprocal translocation between two non-homologues (hypothesis Ib, Fig. S7b). In either case, the second 5S rDNA locus migrated or repositioned distal to the mj-35S rDNA locus.

**Hypothesis-II:** An unequal crossing-over/recombination involving a different chromosome may result in a pericentromeric location of the rDNA loci (35S and the second 5S) in a new variant of *C. mollissima* (Fig. S7c).

**Hypothesis-III:** The NOR (i.e., the mj-35S site) and the second 5S locus were transposed [11] near the pericentromeric location of the same chromosome without any structural rearrangement (inversion or translocation) (Fig. S7d).

**Hypothesis-IV:** Because of behaving as mobile genetic elements [11] the NOR (mj-35S site) including the second 5S may jump to a different homologue in pericentromeric region with no structural modification (Fig. S7e).

**Hypothesis-V:** The NOR (i.e., the mj-35S site) and the satellite including the second 5S locus were repositioned near the centromere of the same chromosome through breakage and fusion (in other words, a shift translocation), in such a scenario there is no need for repositioning of the 5S rDNA site (Fig. S7f) unless the NOR and satellite inverted at the time of fusion.

**Hypothesis-VI:** A breakage – fusion or a segmental reciprocal translocation occurred between two non-homologous chromosomes to create a new *C. mollissima* line like the European accession (Fig. S7g).

Whatever event prevailed, a comparative cyto-molecular study involving European and North American accessions of Chinese chestnut along with other major *Castanea* species using genetically mapped probes [12] is needed to clarify this variation and shed further light on the evolution of this region in these important species.

**Supplementary Figures**

**Supplementary Figure S1: Somatic metaphase chromosome spread of Chinese chestnut CC3 stained with DAPI.**


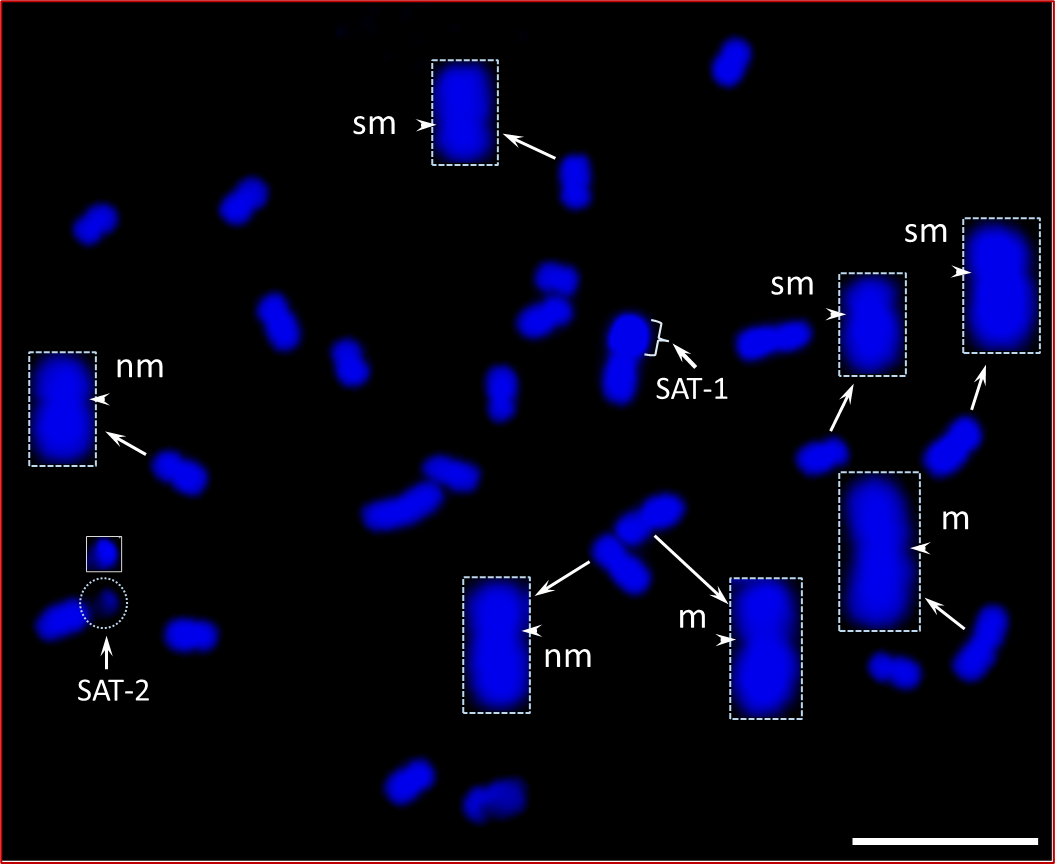


**Figure S1A.** DAPI stained somatic chromosome spread of Chinese chestnut (CC3) displaying chromosomes with different centromeric morphologies, namely metacentric (m), near metacentric (nm) and sub-metacentric (sm). Inserts are the enlarged images of each morphological type. Arrowheads point to the primary constriction (i.e., centromere). Cytomorphological difference in appearance of the satellite pairs: Highly heterochromatic AT-rich SAT-1 (brace) and small euchromatic SAT-2 (lower-left, encircled with white dotted line). An enhanced, enlarged image of SAT-2 is shown above the circle in box. The NOR and satellite can be viewed more clearly in **Figure 7** (an early metaphase spread, main text). Scale = 5 µm.

**Supplementary Figure S1B: Somatic chromosome spreads (mid-prophase, late-prophase and pro-metaphase) stained with Azure-B.**


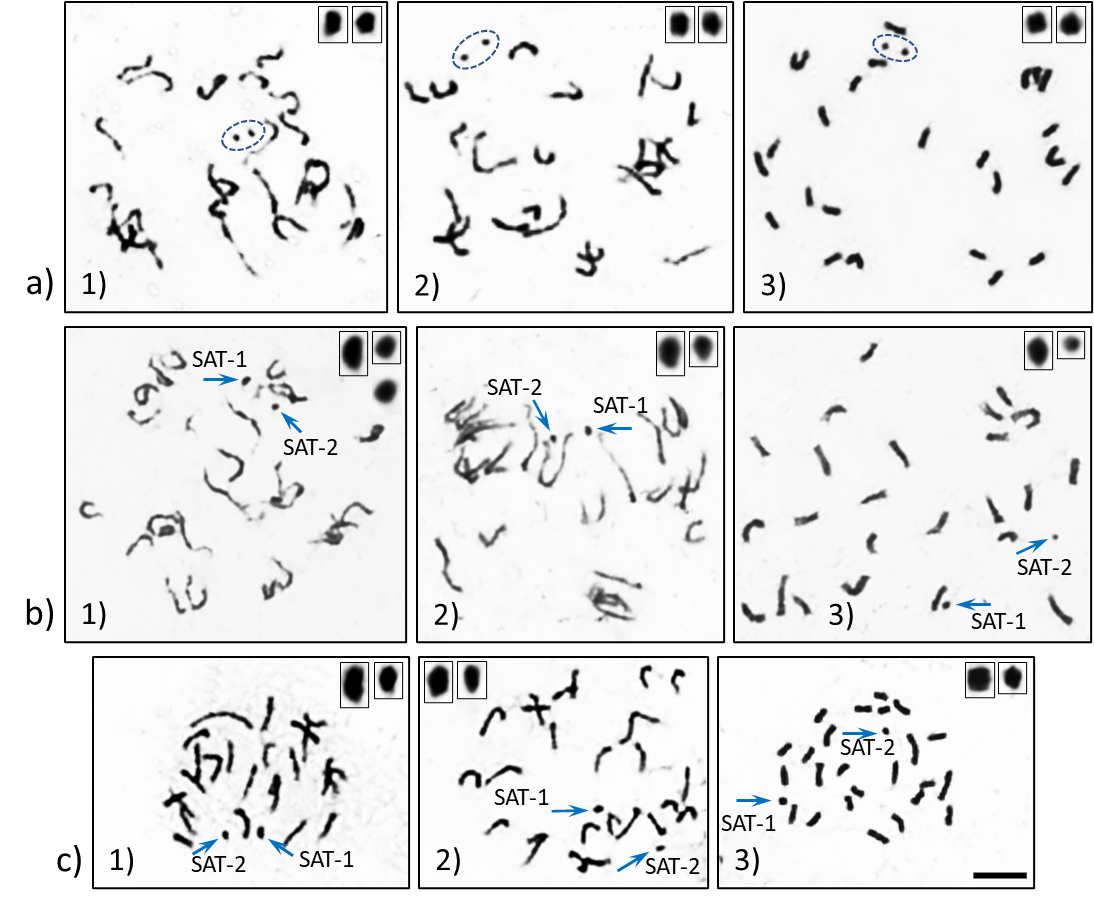


**Figure S1B.** Somatic chromosome spreads of Chinese chestnut accessions (‘a’, CC1, ‘b’, CC3 and ‘c’, CC4) stained with Azure-B, showing the physical size difference of SAT-1 and SAT-2; a1-a3) spreads are in mid-prophase, late-prophase and pro-metaphase stages, respectively, with both satellites (encircled in dotted lines) equal in size; b1-b3) mid-prophase, late-prophase, pro-metaphase stages, respectively, the SAT-1 is significantly larger than the SAT-2; c1-c3) late-prophase, late-prophase and pro-metaphase stages, respectively, the SAT-2 is relatively smaller than the SAT-1. An enlarged image of individual satellites (SAT-1 on the left and SAT-2 on the right) is shown at the top of each chromosome spread. Bar = 5 µm.

**Supplementary Figure S2: FISH with 35S and 5S rDNA probes of prophase and pro-metaphase chromosomes of American chestnut and Chinese chestnut.**


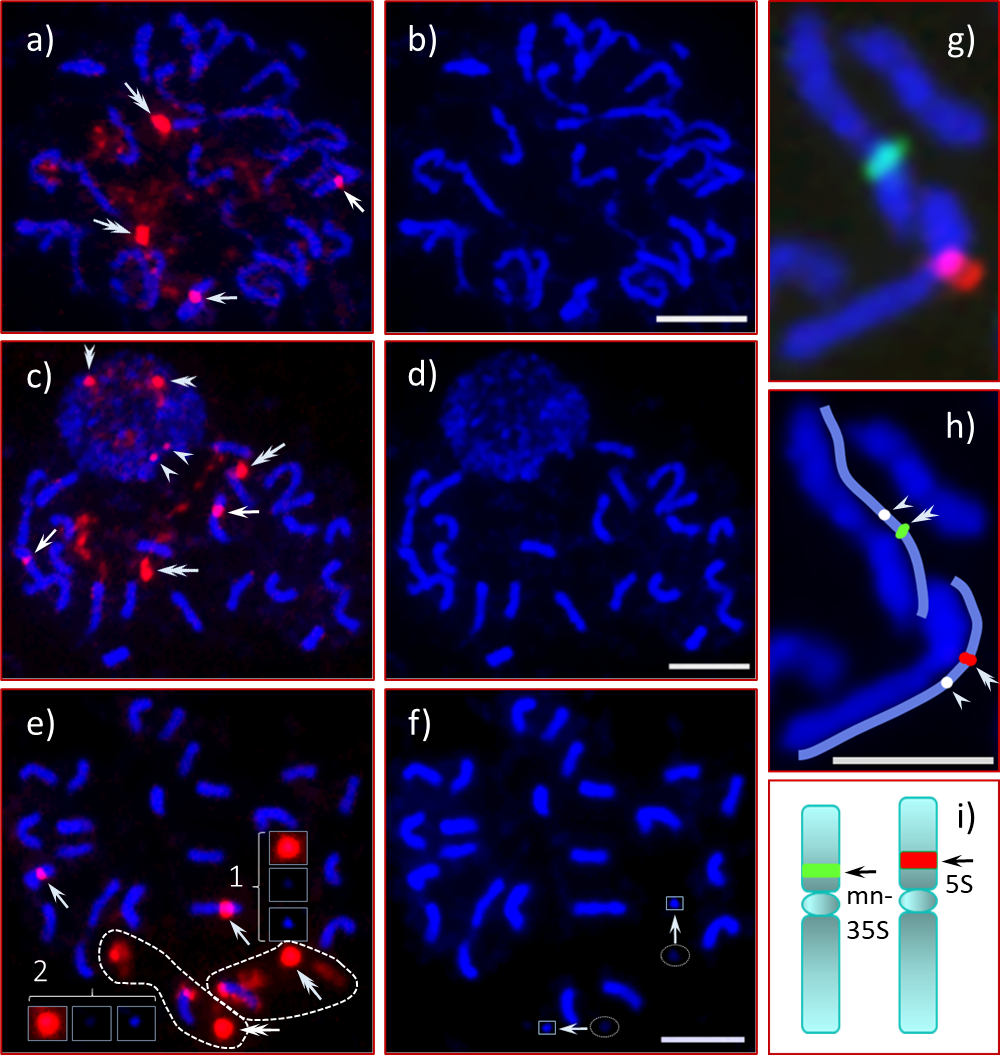


**Figure S2.** American chestnut chromosome spreads at mid-prophase (**a**), late-prophase (**c**) and pro-metaphase (**e**) hybridized with the 35S rDNA probe (red signals) and counter stained with DAPI. **a**) shows two large and two smaller bodies of the 35S signal along with numerous scattered signals (red); the large bodies (red signals, double arrows) with similar intensities are from the mj-35S, while the medium-sized bodies (arrows) with different intensities are from the mn-35S locus; **b**) DAPI stained chromosomes, same cell as in ‘**a**’; **c**) shows two cells at different stages in the cell cycle, a late-prophase cell and an interphase nucleus (top-left); the interphase nucleus showed two large (double arrowheads), two minor (arrowheads) 35S signals along with numerous scattered red 35S signals, the description for the late prophase cell is same as ‘**a’**; **d**) DAPI stained chromosomes and the interphase nucleus are same as in ‘**c**’; **e**) a pro-metaphase spread, the individual mother chromosomes with the mj-35S rDNA signals (red signals shown in dotted circles), a solid heavy terminal signal appeared as a distinct ball completely covering the respective satellite. The top in the panel ‘**e1**’ (middle-right) is the satellite that is completely covered by the 35S signal, insert in the middle of the same panel shows a faint DAPI staining satellite (almost invisible), but the bottom insert of the same panel shows a DAPI stained chromosome body (image was enhanced further) i.e., the satellite; description for the panel ‘**e2**’ (from left to right) is same as in panel ‘**e1**’; **f**) DAPI stained chromosomes, same cell as in “**e**”, shows the satellites of normal intensity (dotted oval circles) and of greater intensity – enhanced further (arrows, dotted rectangular areas). An enlarged FISH image of a portion of Chinese chestnut mn-35S (green signal) and 5S (red signal) bearing chromosomes given in ‘**g**’ (observed as a pair of signals, one on each chromatid), a light blue line over each of these chromosomes (**h**) shows the centromere (arrowhead, white) and FISH signal positions (double arrowheads – green or red), diagrammatic illustration is given in ‘**i**’. Bars = 5 µm (**f**), and 2.5 µm (**h**).

**Supplementary Figure S3: Organization of the mj-35S rRNA and structural details of the major 35S rDNA bearing chromosome in American chestnut.**


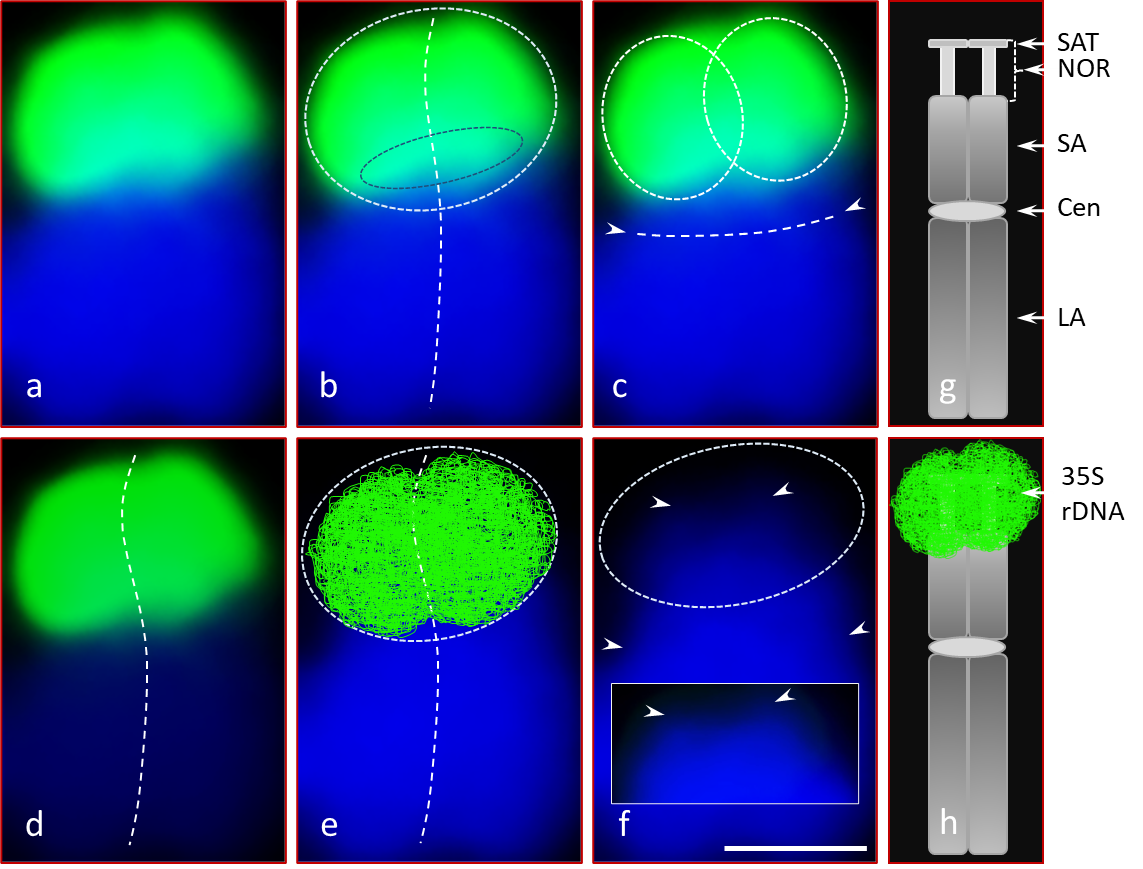


**Figure S3.** Enlarged image of one of the mj-35S rDNA bearing chromosomes of American chestnut (see **Fig 1d**, main text) is shown in ‘**a**’ and image processed further (‘**b**’ to ‘**f**’) including diagrammatic illustrations to demonstrate the organizational composition and distribution of the mj-35S rRNA gene and the structural insights of the chromosome in respect to chromatids (sister chromosomes), short arm, NOR, and the details of the 35S FISH signal. **b**) The mj-35S signal encircled with white dotted line, the distal end of the short arm encircled with oval shaped blue dotted line, longitudinal white dotted line drawn along the demarcation line between two chromatids (weak DAPI line that separates the chromatids, compare with image in ‘**a**’); **c**) two lobes of 35S signals (oval shaped white dotted circles), one on each chromatid, the centromere marked by a crossed white dotted line and arrowheads (compare with images in ‘**a**’ and/or ‘**b**’); **d**) image with reduced DAPI (processed further) enhances the 35S green signal and the brightness appears to be uniform, clearly shows two sharp dents (top and bottom of the signal) that demonstrate each lobe of signal on each chromatid marked by a longitudinal white dotted line; **e**) two oval roundish strings of lines drawn over each lobe (looks like two woolen balls, encircled with white dotted line) that represent the very high copy number of rRNA gene; **f**) a DAPI image of the chromosome, arrowheads (top) marked the terminal end (satellite’s end), and primary constriction (centromere region) marked by arrowheads (just below the oval shaped white dotted line), insert (rectangular box, bottom) enhanced DAPI image clearly shows the chromosome end of the short arm (arrowheads); **g**) a diagrammatic illustration of the chromosome (SAT = Satellite, NOR = Nucleolus Organizing Region, SA = Short arm, Cen = Centromere, LA = Long arm); **h**) same illustration as ‘**g**’ shows two lobes of 35S rRNA genes (spiral ball of green signal). Bars = 2.5 µm (**h**).

**Supplementary Figure S4: Chinese chestnut CC3 rDNA associated satellites.**


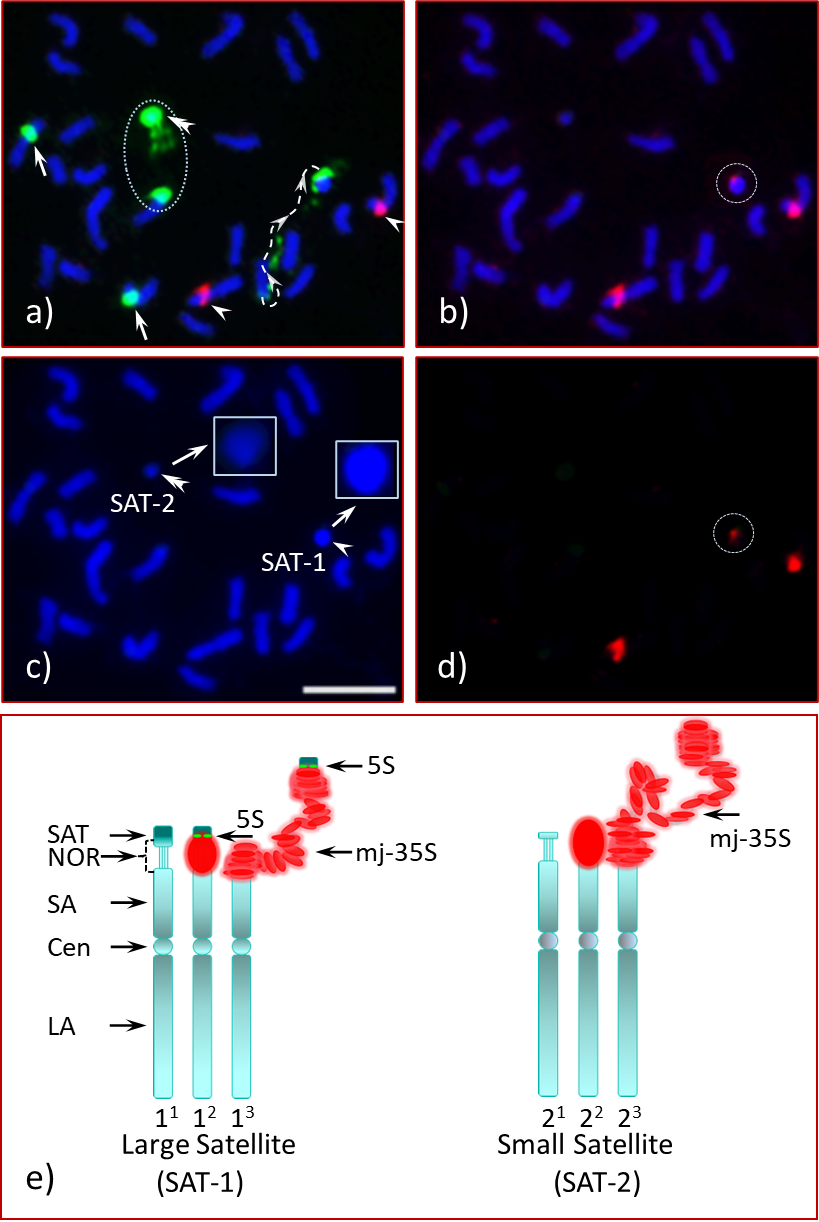


**Figure S4.** Chinese chestnut CC3 root tip chromosome spread (pro-metaphase) hybridized with 35S (green signals) and 5S (red signals) rDNA probes; **a**) arrows and arrowheads point at the mn-35S and 5S rDNA signals, respectively. For the major 35S pair, the proximal half of the SAT-1 is covered by the signal, a white dotted line along with arrowheads drawn over the scattered 35S signal, the path of the NOR, that connects the mother chromosome; SAT-2 completely covered by the 35S signal (double arrowheads) and the end of the short arm (including the signal) of the mother chromosome is encircled with white dotted line (middle-center); **b**) same image as in ‘**a**’ under red-blue filters to clearly mark the second 5S site (encircled with white dotted line); **c**) same cell as in ‘**a**’ under blue filter (DAPI image), SAT-1 and SAT-2 marked as arrowhead and double arrowheads respectively, enlarged image of the same shown in boxes; **d**) same cell as in ‘**a**’ under red filter to show all the 5S signals including the second site (circle); **e**) diagrammatic illustration of the mj-35S rDNA bearing homologues, **e1^1^**) structural outline of the SAT-1 homologue (abbreviations are the same as in **Fig. S3**), **e1^2^**) shows the position of the mj-35S locus (NOR) covering the end of the short arm, the secondary constriction and the proximal half of the satellite, the second 5S (green signal) positioned in the distal region of the mj-35S signal (red), **e1^3^**) structural format of a late prophase or pro-metaphase chromosome with dispersed mj-35S signals (red) and the satellite showed the second 5S signal (green); **e2^1-3^**) description is same as ‘**e1^1-3^**’ except it does not contain the second 5S site, and the satellite (SAT-2) covered completely by the mj-35S signals. Bar = 5µm.

**Supplementary Figure S5: The major rDNA distribution in Chinese chestnut CC3-- NOR and its satellite in a mid/late-prophase chromosome and SAT-2 in a pro-metaphase chromosome.**


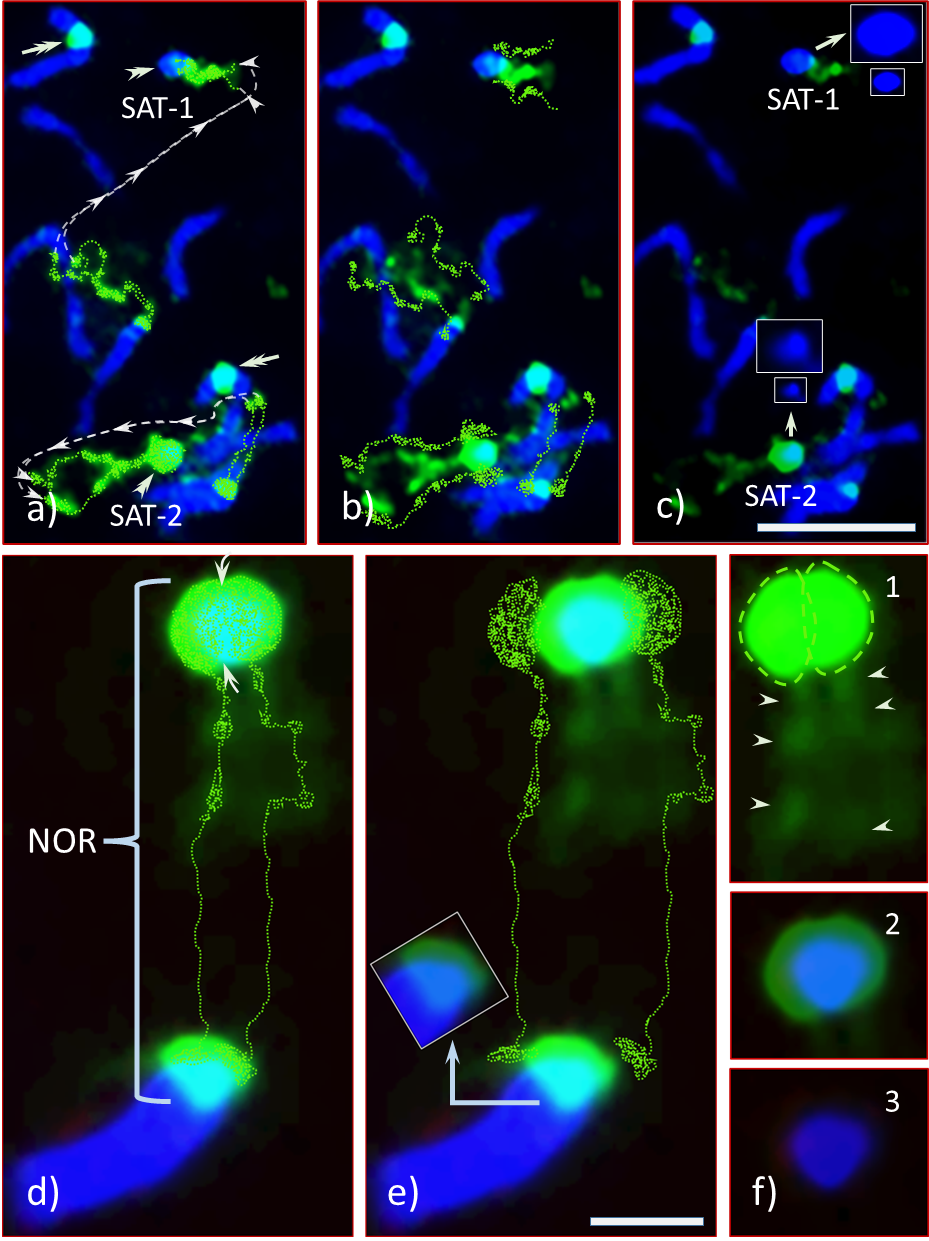


**Figure S5.** Shows a portion of Chinese chestnut CC3 mid-prophase (**a**, **b** and **c**) and an enlarged image of SAT-2 rDNA bearing pro-metaphase chromosome of Figure S4a shown in **d** and **e** along with mj-35S signals (green). **a**) The mn-35S always observed to be intact with the respective mother chromosome (double arrows) in prophase to pro-metaphase stages [see **Figs. 2-4** (main text), **Figs. S2** and **S4**)], there is no visible sign of NOR in this region probably because of much reduced copy number of the 35S rDNA unit when compared to mj-35S and/or the NOR is much shorter for this mn-35S site; double arrowheads mark the respective mj-35S rDNA mother chromosome’s SAT-1 and SAT-2, and observed scattered mj-35S signals of various sizes [also see **Figs. 2** and **3** (main text); **Figs. S2** and **S4**] seen near the vicinity of these chromosomes. Various sizes (small-medium-large) of rDNA signals might be due to the flexible Inter-Genic-Spacer (IGS) length or various degree of contraction/condensation at NOR as postulated in **Fig. S6** (see below). The mj-35S signals that spread over the NOR clearly show two strands as drawn green dotted lines over the signals, which represent two chromatids of the chromosome, white dotted lines along with arrowheads show the path of the NOR connection between the respective mother chromosome with its satellite; **b**) each green dotted line represents individual chromatid’s (NOR) path beside the FISH signals; **c**) same spread as in ‘**a**’ with reduced (or weak) green signal (image processed further) show the blended effect of SAT-2’s green and a faint proximal green signal of SAT-1, inserts are the respective satellites, but for SAT-2 the smaller box shows the original image that was completely masked by 35S green signal, the larger box of each satellite shows an enlarged image of the same (image processed further to enhance DAPI); **d**) an enlarged image of SAT-2 pro-metaphase chromosome (see **Fig. S4a**), green dots that represent the path of the 35S rRNA gene drawn over the green mj-35S signal including two lobes (one on each chromatid) marked by two bent arrows show the partition point of the lobes; **e**) the green dotted lines depicting the 35S rRNA gene shown along the side of the two lobes and the secondary constriction represent the chromatids at the NOR, demonstrating the rDNA organization within NOR (including the end of the short arm, secondary constriction and the satellite). A further processed image in the lower-left insert shows dim (or pale) green mj-35S signal and a blended effect of the DAPI from the distal short arm; f) three different processed images of the SAT-2 shown in panels f1, f2 and f3; **f1**) through image process removed the DAPI stain leaving only the bright greenish FISH signal and each lobe composed of numerous copies of 35S rRNA gene encircled with greenish dotted lines and each lobe is connected with the respective chromatid of the NOR as shown by two strings of light greenish signals (arrowheads), **f2**) reduced green signal exposes the blended DAPI image of the satellite (SAT-2), and **f3**) shows the DAPI stained satellite without green signal. Bars = 5 µm (**c**), and = 1 µm (**e**).

**Supplementary Figure S6: FISH-based schematic model of 35S rDNA and NOR condensation.**


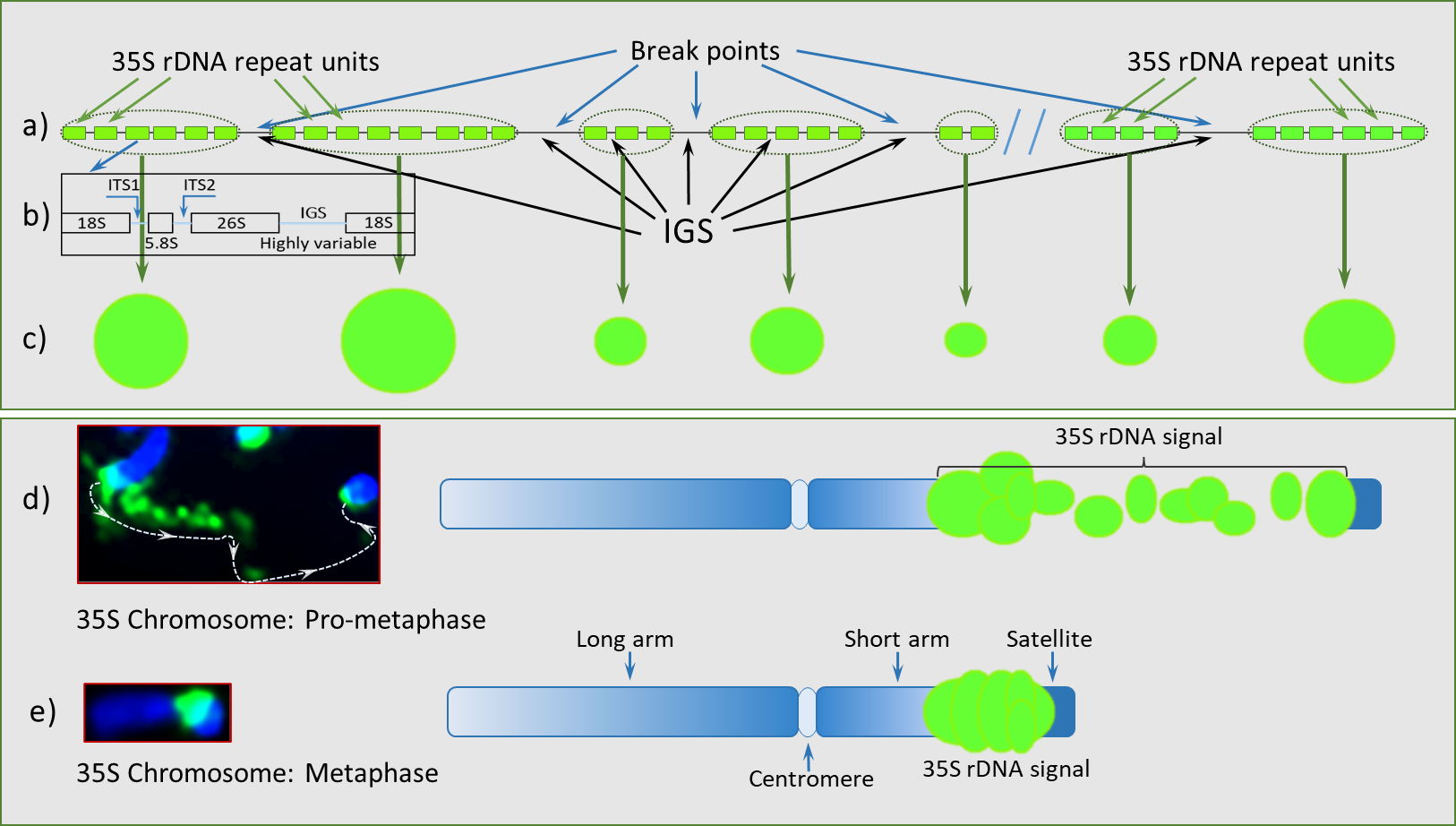


**Figure S6.** A diagrammatic sketch of the structure of 35S rDNA unit shows numerous repeat units with variable IGS (intergenic spacer) that yield varying sizes of FISH signals. As the DNA condensation progresses the signal sizes get bigger and the number of units reduced ultimately forming a single prominent signal; **a**) 35S rDNA units with variable ISG on nucleolus organizing region (NOR) in interphase nucleus; **b**) 35S rDNA unit composed of 18S-ITS1-5.8S-ITS2-26S, and each 35S unit separated by an IGS; **c**) There are seven clusters of 35S rDNA repeat units forming various sizes of signal (small to large) depending on the number of 35S rDNA repeat units in each cluster. These clusters may get separated during chromosome preparation thus resulting in multiple FISH signals (various intensities) on the spreads; **d**) a pro-metaphase mj-35S rDNA bearing chromosome including a satellite (it is SAT-1, see **Fig. 3b,** main text) with a number of concentrated, but dispersed 35S rDNA signals, a white dotted line with arrowheads drawn along the path of the signals (it is the NOR region of the chromosome) that connect the satellite, a diagrammatic illustration is given next to it; **e**) an early metaphase chromosome (see **Fig. 3d,** main text) with one aggregated (large) signal, a diagrammatic illustration also shown next to it.

**Supplementary Figure S7: Diagrammatic Illustration of Structural Rearrangement.**

**Supplementary Figure S7a: A whole arm inversion from either Chinese chestnut CC3 or CC4.**


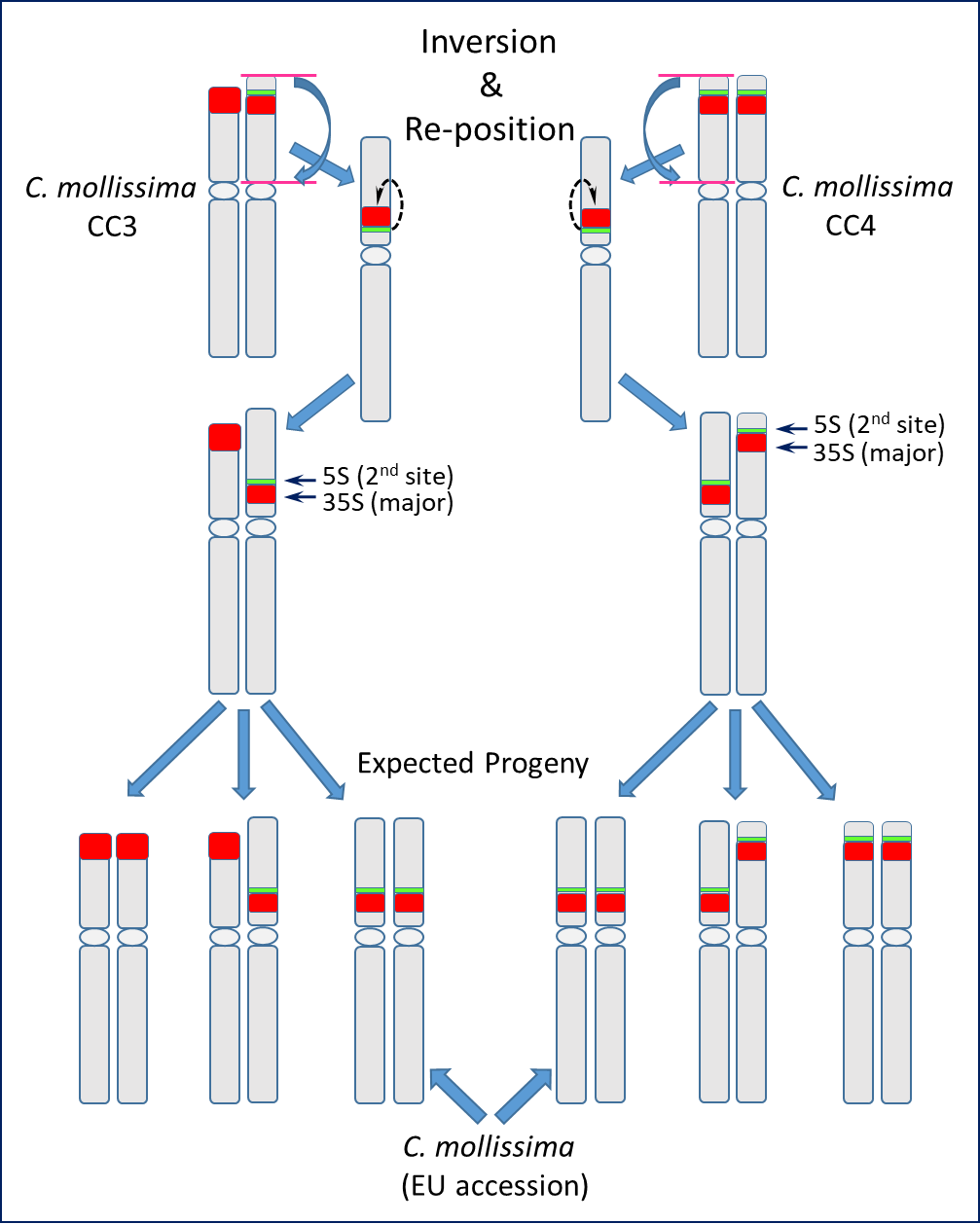


**Figure S7a.** A whole arm inversion from either CC3 or CC4 or a similar accession of *C. mollissima*, and then migrated the 5S distal to the mj-35S rDNA locus to create a new *C. mollissima* line like the European accession.

**Supplementary Figure S7b: Whole arm inversion from either Chinese chestnut CC3 or CC4 followed by reciprocal translocation.**


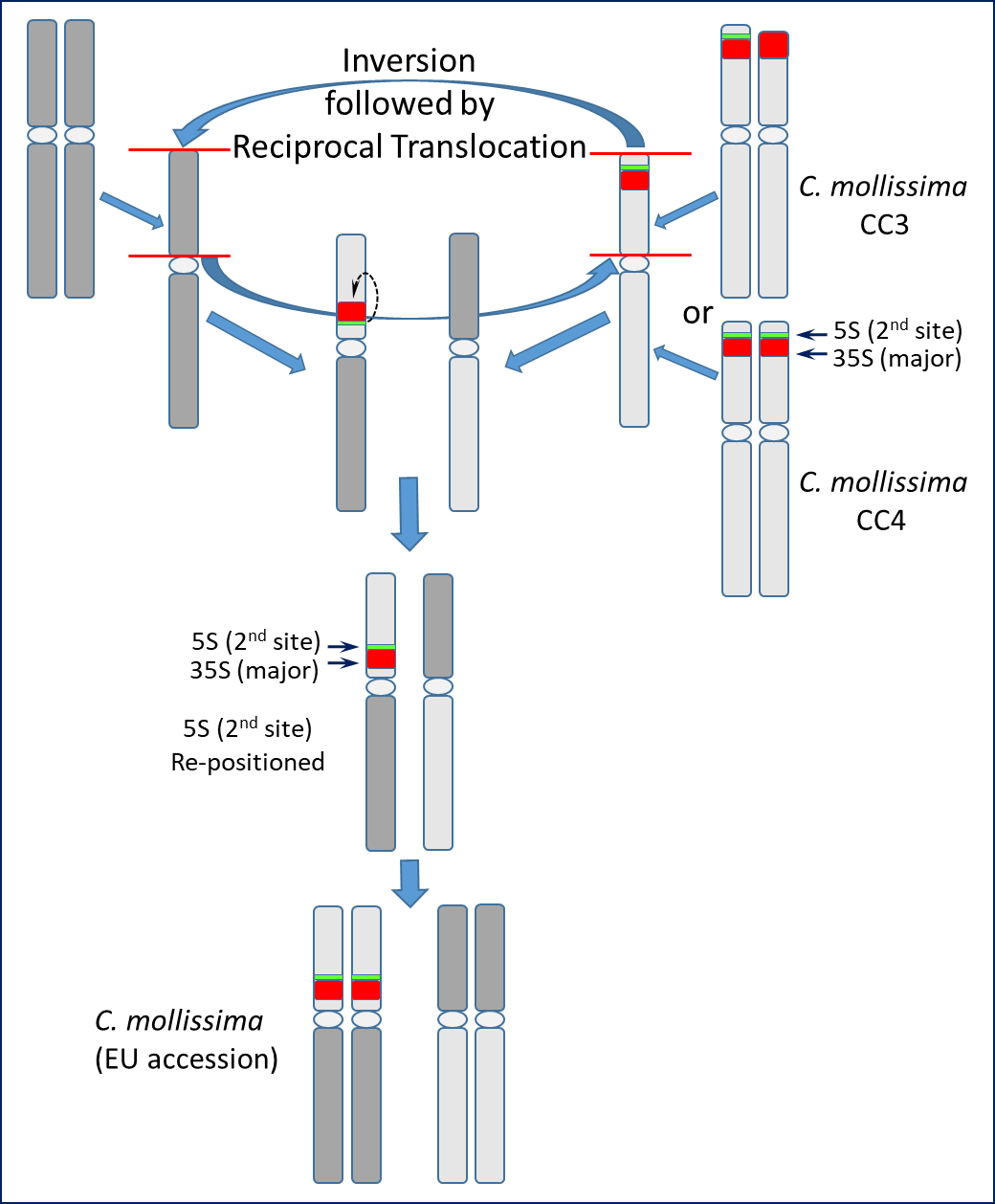


**Figure S7b.** Whole arm inversion followed by a reciprocal translocation between two non-homologous chromosomes, and then migrated the 5S distal to the mj-35S rDNA locus from either CC3 or CC4 or a similar accession of *C. mollissima* to create a new *C. mollissima* line like the European accession.

**Supplementary Figure S7c: An unequal crossing over/recombination with a different chromosome.**


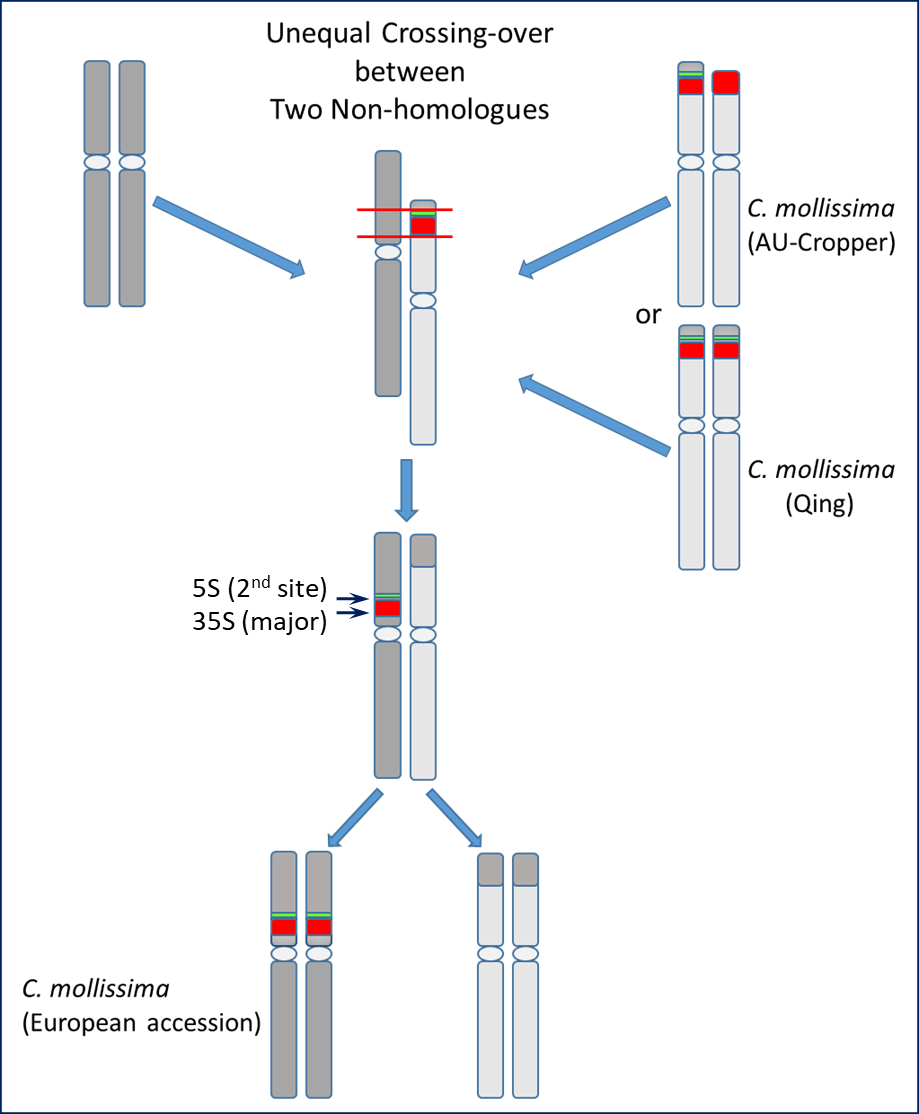


**Figure S7c.** An unequal crossing over between two non-homologous chromosomes may result pericentromeric location the rDNA loci (mj-35S and the second 5S) to create a new *C. mollissima* line like the European accession.

**Supplementary Figure S7d: Repositioning of the major 35S and the second 5S rDNA loci.**

**
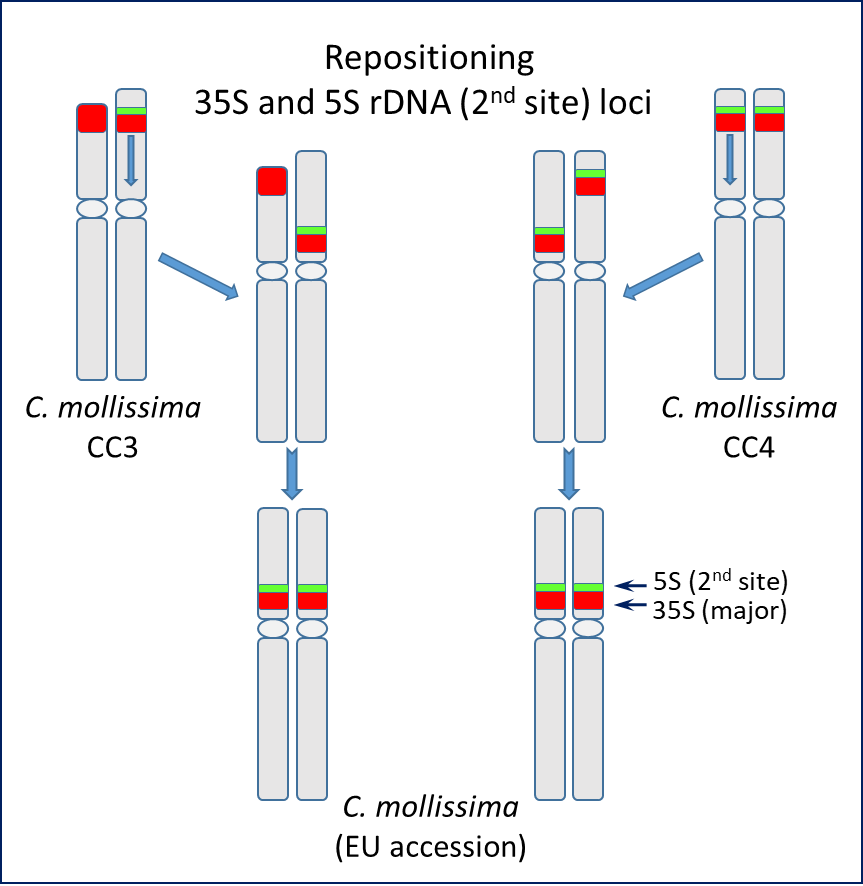
**

**Figure S7d.** Diagrammatic representation of repositioning of the mj-35S and 5S rDNA loci in either CC3 or CC4 or a similar accession of *C. mollissima* to create a new *C. mollissima* line like the European accession.

**Supplementary Figure S7e: Major 35S and the second 5S rDNA loci moved to a non-homologue.**


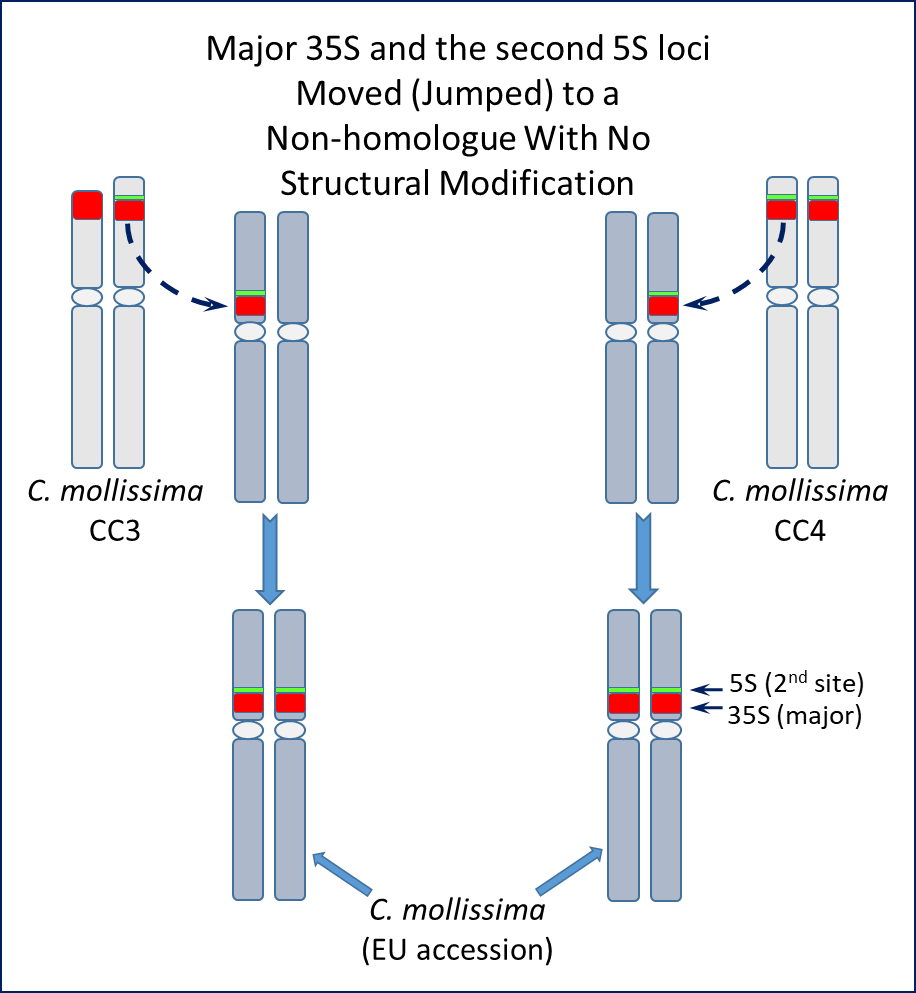


**Figure S7e.** Diagrammatic representation of repositioning like mobile elements the mj-35S and the second 5S rDNA loci from either CC3 or CC4 or a similar accession of *C. mollissima* through moving (jumping) to another to create a new *C. mollissima* line like the European accession.

**Supplementary Figure S7f: Breakage – fusion or shift translocation.**


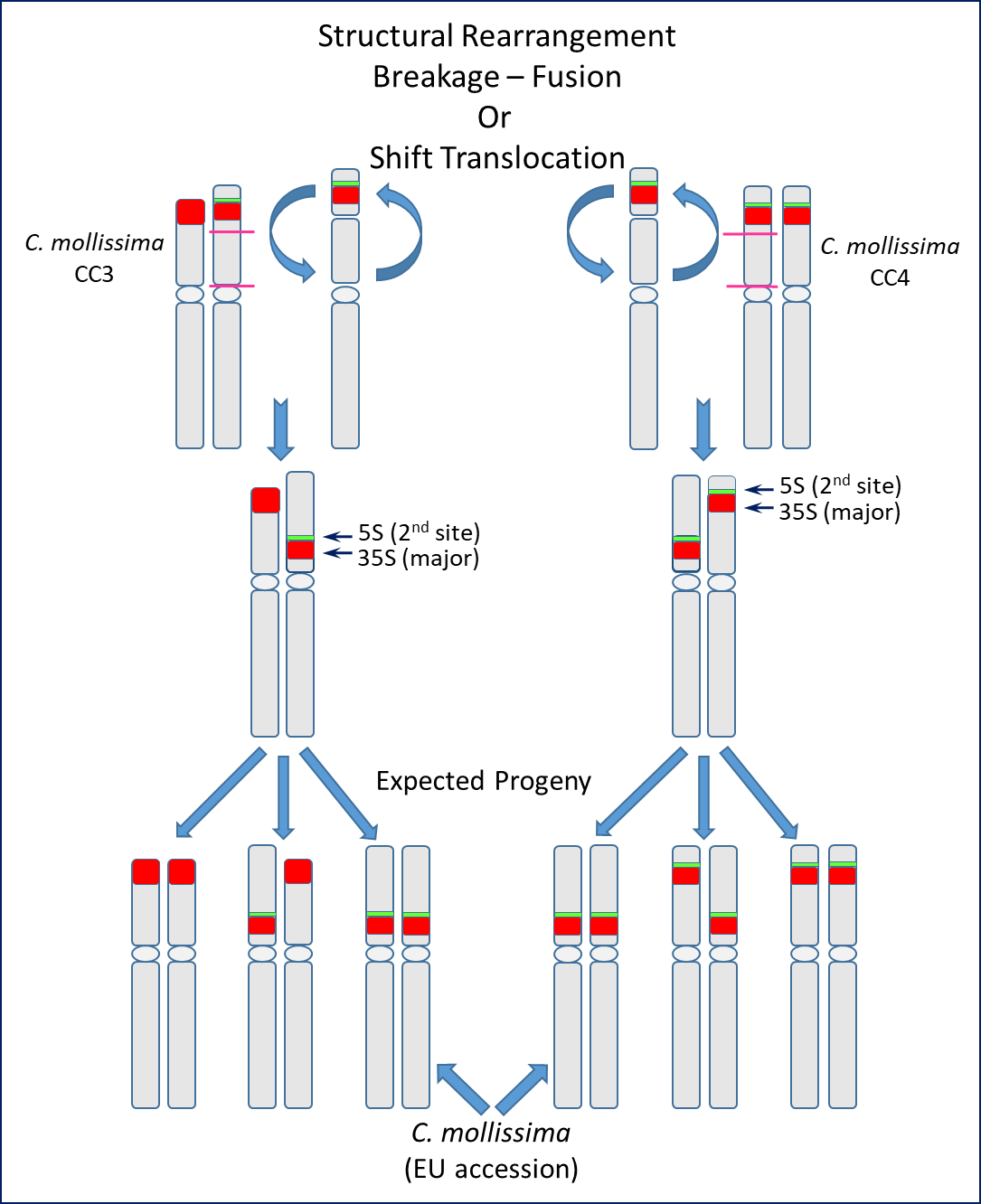


**Figure S7f.** Diagrammatic representation of structural rearrangement through Breakage-Fusion event involving the mj-35S and 5S rDNA loci in either CC3 or CC4 or a similar accession of *C. mollissima* to create a new *C. mollissima* line like the European accession.

**Supplementary Figure S7g: Breakage–fusion between two non-homologues.**


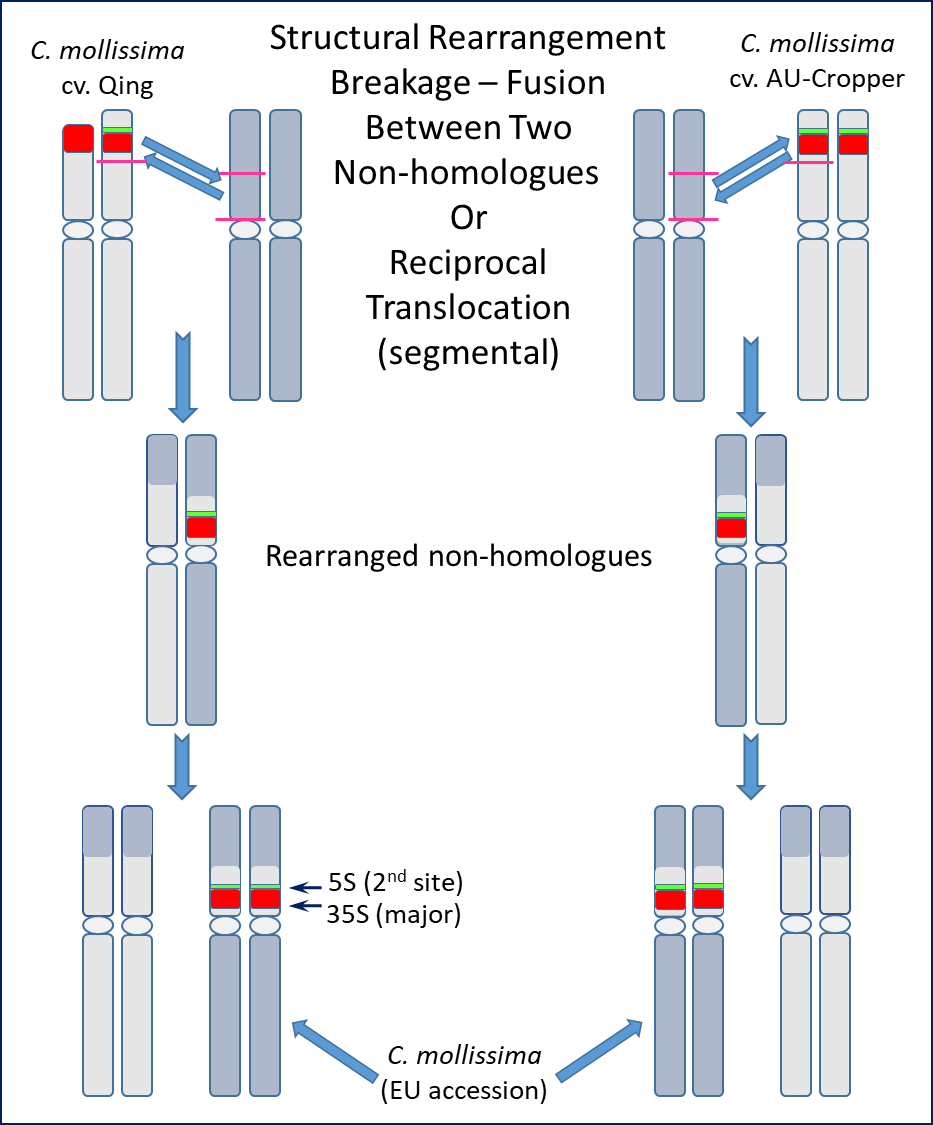


**Figure S7g.** Diagrammatic representation of structural rearrangement through Breakage-Fusion event between two non-homologues or a segmental reciprocal translocation involving the mj-35S and 5S rDNA loci in either CC3 or CC4 or a similar accession of *C. mollissima* to create a new *C. mollissima* line like the European accession.

**References**

1. Ribeiro, T., Loureiro, J., Santos, C., and Morais-Cecílio, L. Evolution of rDNA FISH patterns in the Fagaceae. Tree Genetics & Genomes 7:1113-1122. 10.1007/s11295-011-0399-x (2011).
2. Laemmli, U. K. et al. Metaphase chromosome structure: The role of nonhistone proteins. In Cold Spring Harb. Symp. Quant. Biol. Vol. 42, 351-360 (cold Spring Harbor Laboratory Press, 1978). 10.1101/sqb.1978.042.01.036 (1978).
3. Garcia, S. *et al.* Linkage of 35S and 5S rRNA genes in *Artemisia* (family Asteraceae): first evidence from angiosperms. *Chromosoma* **118,** 85–97; 10.1007/s00412-008-0179-z (2009).
4. Poczai, P., & Hyvönen, J. Nuclear ribosomal spacer regions in plant phylogenetics: problems and prospects. Molec. Biol. Rep. 37, 1897-1912. 10.1007/s1133-009-9630-3 (2010).
5. Zoldos, V. et al. Molecular-cytogenetic studies of ribosomal genes and heterochromatin reveal conserved genome organization among 11 *Quercus* species. Theor. Appl. Genet. **99,** 969-977. 10.1007/s001220051404 (1999).
6. Islam-Faridi, M. N., Nelson, C. D., DiFazio, S. P., Gunter, L. E. & Tuskan, G. A. Cytogenetic analysis of *Populus* *trichocarpa* – ribosomal DNA, telomere sequence, and marker-selected BACs. Cytogenet. Genome Res. **125,** 74-80. 10.1159/000218749 (2009).
7. Islam-Faridi, N., Sakhanokho, H. F., & Nelson, C. D. New chromosome number and cyto-molecular characterization of the African baobab (*Adansonia* *digitata* L.) – “The Tree of Life”. Scientific Reports 10, 13174. 10.1038/s41598-020-68697-6 (2020).
8. Cerbah, M., Coulaud, J., & Siljak-Yakovlev S. rDNA organization and evolutionary relationships in the genus *Hypochaeris* (Asteraceae). J. Hered. 89, 312-318 (1998).
9. Lysak, M. A., Berr, A., Pecinka, A., Schmidt, R., McBreen, K., & Schubert, I. Mechanisms of chromosome number reduction in *Arabidopsis thaliana* and related Brassicaceae species. Proc. Natl. Acad. Sci. USA. 103(13), 5224-5229. 10.1073/pnas.0510791103 (2006).
10. Schubert, I. Chromosome evolution. Curr. Opin. Plant Biol. 10, 109-115. 10.1016/j.pbi.2007.01.001 (2007).
11. Schubert, I. Mobile nucleolus organizing regions (NORs) in *Allium* (Liliaceae s. lat.)? – Inferences from the specifity of silver staining. Pl. Syst. Evol. 144 291-305 (1984).
12. Staton, M. et al. A reference genome assembly and adaptive trait analysis of *Castanea* *mollissima* ‘Vanuxem’ a source of resistance to chestnut blight in restoration breeding. *Tree Genetics & Genomes* **16,** 57;10.1007/s11295-020-01454-y (2020).
