# Supplementary material for: Cyto-molecular characterization of rDNA and chromatin composition in the NOR-associated satellite in Chestnut (Castanea spp.)
Source: Sci Rep. 2024 Jan 15;14:980. doi: 10.1038/s41598-023-45879-6 (PMC10789788; doi:10.1038/s41598-023-45879-6)
Supplement: Supplementary file 2 — Supplementary Information 2. [file 41598_2023_45879_MOESM2_ESM.pptx]

## Slide 1
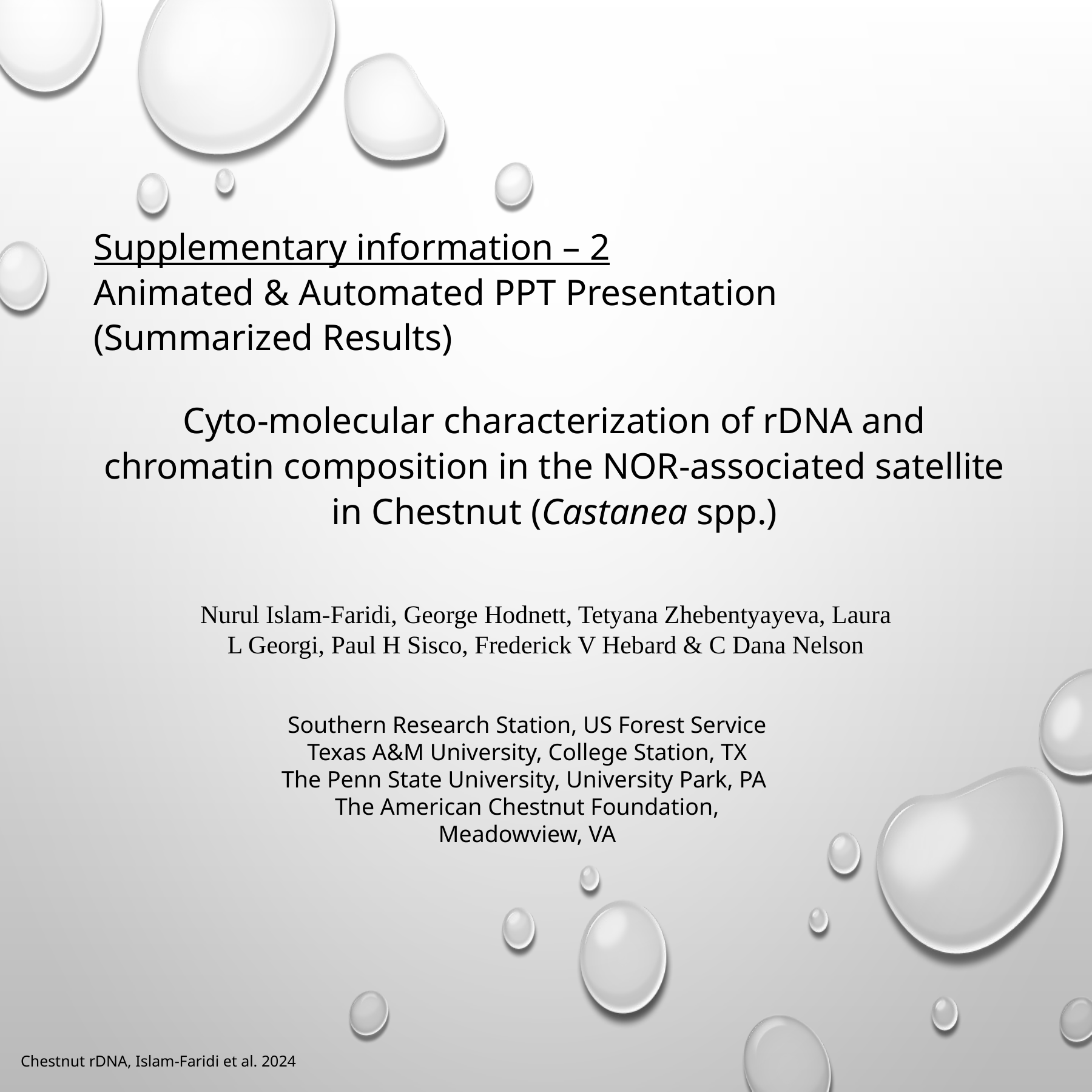

Supplementary information – 2
Animated & Automated PPT Presentation (Summarized Results)
Cyto-molecular characterization of rDNA and chromatin composition in the NOR-associated satellite in Chestnut (Castanea spp.)
Nurul Islam-Faridi, George Hodnett, Tetyana Zhebentyayeva, Laura L Georgi, Paul H Sisco, Frederick V Hebard & C Dana Nelson
Southern Research Station, US Forest Service
Texas A&M University, College Station, TX
The Penn State University, University Park, PA
The American Chestnut Foundation, Meadowview, VA
Chestnut rDNA, Islam-Faridi et al. 2024

## Slide 2
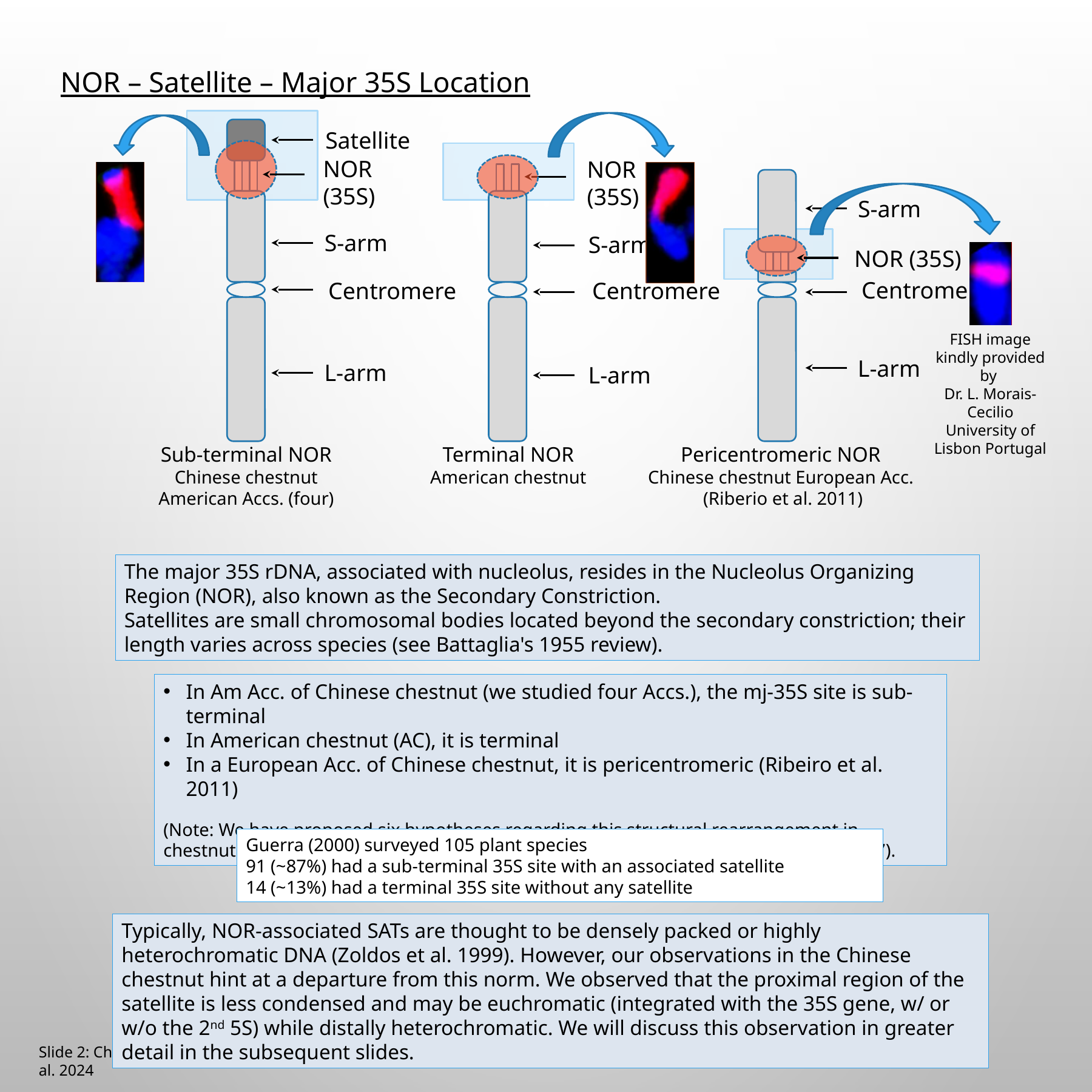

NOR – Satellite – Major 35S Location
Satellite
NOR
(35S)
NOR
(35S)
S-arm
S-arm
S-arm
NOR (35S)
Centromere
Centromere
Centromere
FISH image kindly provided by
Dr. L. Morais-Cecilio
University of Lisbon Portugal
L-arm
L-arm
L-arm
Sub-terminal NOR
Chinese chestnut
American Accs. (four)
Terminal NOR
American chestnut
Pericentromeric NOR
Chinese chestnut European Acc.
 (Riberio et al. 2011)
The major 35S rDNA, associated with nucleolus, resides in the Nucleolus Organizing Region (NOR), also known as the Secondary Constriction.
Satellites are small chromosomal bodies located beyond the secondary constriction; their length varies across species (see Battaglia's 1955 review).
In Am Acc. of Chinese chestnut (we studied four Accs.), the mj-35S site is sub-terminal
In American chestnut (AC), it is terminal
In a European Acc. of Chinese chestnut, it is pericentromeric (Ribeiro et al. 2011)
(Note: We have proposed six hypotheses regarding this structural rearrangement in chestnuts. For details see the Supplementary information 1, and Supplementary Fig. S7).
Guerra (2000) surveyed 105 plant species
91 (~87%) had a sub-terminal 35S site with an associated satellite
14 (~13%) had a terminal 35S site without any satellite
Typically, NOR-associated SATs are thought to be densely packed or highly heterochromatic DNA (Zoldos et al. 1999). However, our observations in the Chinese chestnut hint at a departure from this norm. We observed that the proximal region of the satellite is less condensed and may be euchromatic (integrated with the 35S gene, w/ or w/o the 2nd 5S) while distally heterochromatic. We will discuss this observation in greater detail in the subsequent slides.
Slide 2: Chestnut rDNA, Islam-Faridi et al. 2024

## Slide 3
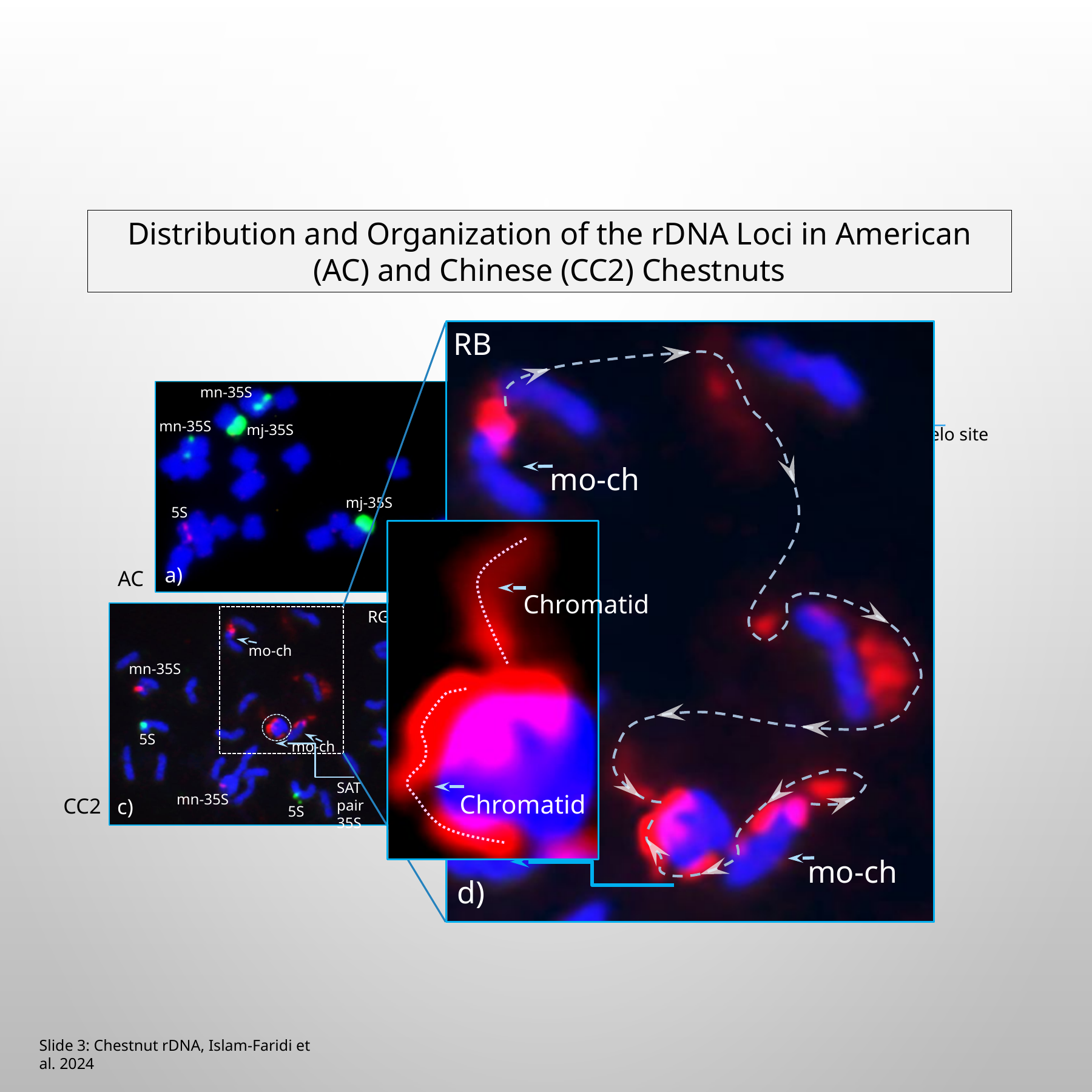

Distribution and Organization of the rDNA Loci in American (AC) and Chinese (CC2) Chestnuts
RB
mn-35S
RGB
B
SAT
mn-35S
mj-35S
Telo site
mo-ch
mj-35S
5S
5S
b)
a)
5µm
AC
Chromatid
RGB
B
Satellite
pair
mo-ch
mn-35S
5S
mo-ch
SAT pair
35S
Chromatid
mn-35S
CC2
c)
e)
5µm
5S
mo-ch
d)
Slide 3: Chestnut rDNA, Islam-Faridi et al. 2024

## Slide 4
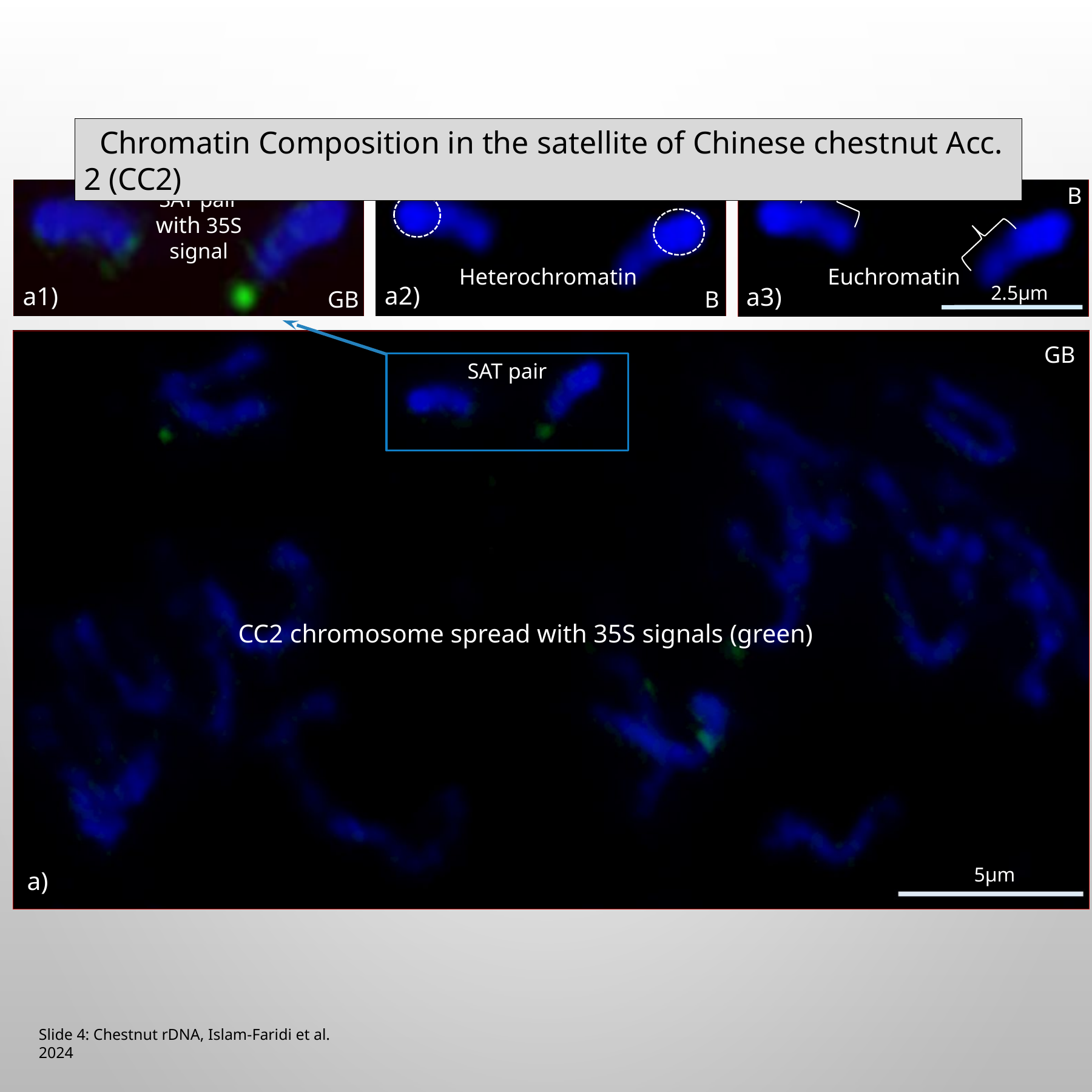

Chromatin Composition in the satellite of Chinese chestnut Acc. 2 (CC2)
B
SAT pair
with 35S
signal
Heterochromatin
Euchromatin
a2)
a1)
2.5µm
a3)
GB
B
GB
SAT pair
CC2 chromosome spread with 35S signals (green)
5µm
a)
Slide 4: Chestnut rDNA, Islam-Faridi et al. 2024

## Slide 5
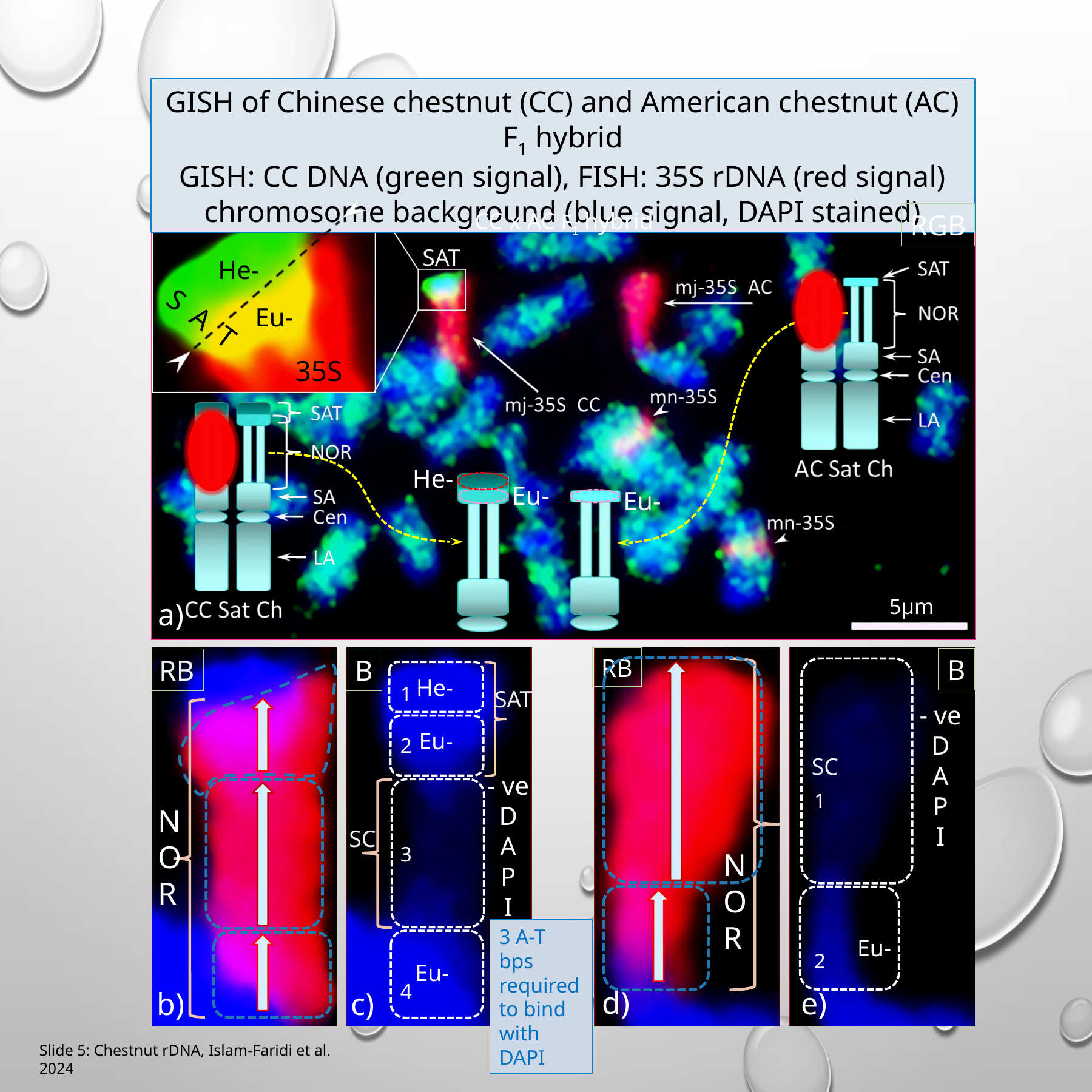

GISH of Chinese chestnut (CC) and American chestnut (AC) F1 hybrid
GISH: CC DNA (green signal), FISH: 35S rDNA (red signal)
chromosome background (blue signal, DAPI stained)
CC x AC F1 hybrid
RGB
SAT
He-
S A T
Eu-
35S
He-
Eu-
Eu-
5µm
a)
RB
B
RB
B
He-
1
SAT
- ve
D
A
P
I
Eu-
2
SC
- ve
D
A
P
I
1
N
O
R
SC
3
N
O
R
3 A-T bps
required to bind with DAPI
Eu-
2
Eu-
4
d)
e)
b)
c)
Slide 5: Chestnut rDNA, Islam-Faridi et al. 2024

## Slide 6
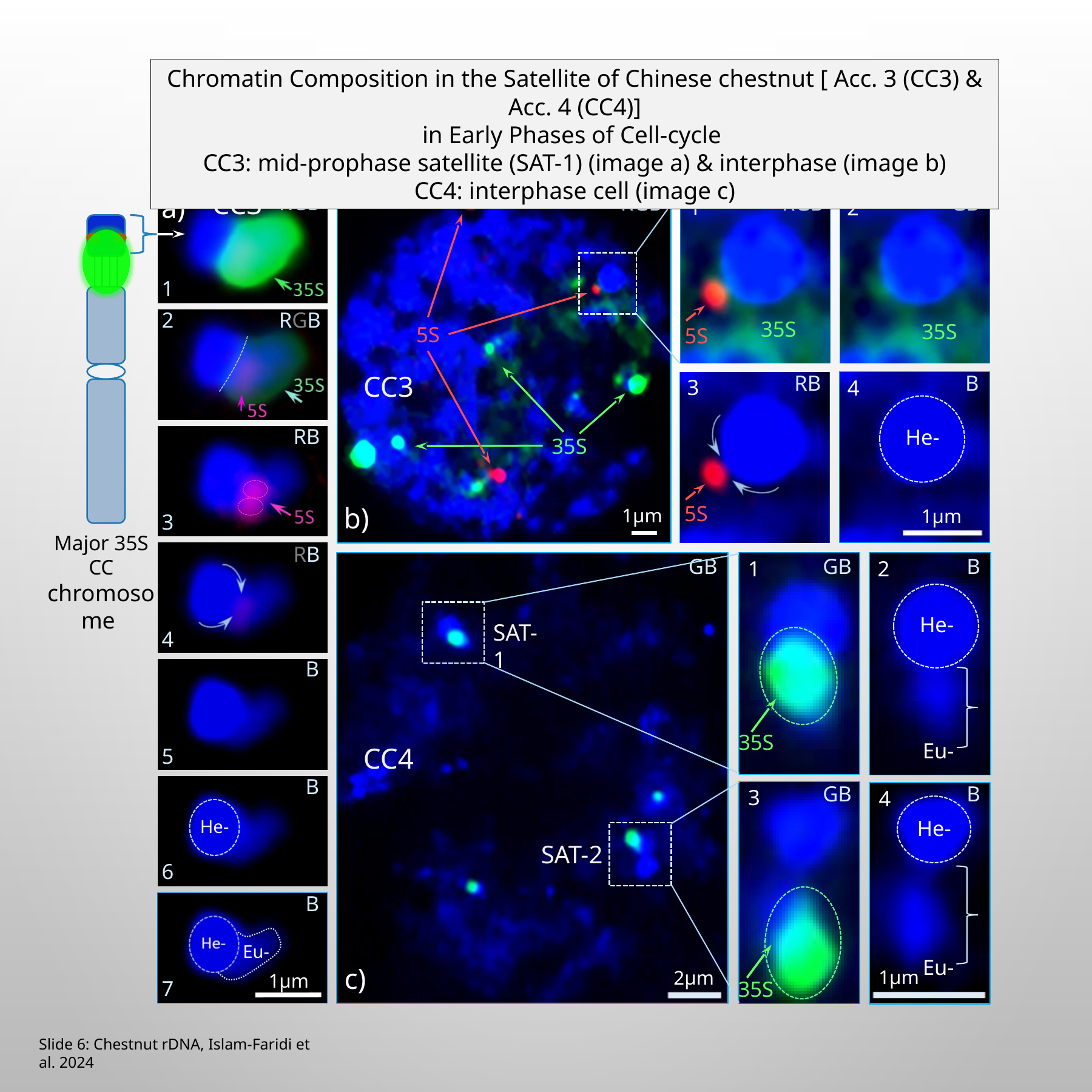

Chromatin Composition in the Satellite of Chinese chestnut [ Acc. 3 (CC3) & Acc. 4 (CC4)]
in Early Phases of Cell-cycle
CC3: mid-prophase satellite (SAT-1) (image a) & interphase (image b)
CC4: interphase cell (image c)
CC3
RGB
RGB
RGB
GB
a)
1
2
1
RGB
2
35S
35S
5S
5S
CC3
RB
B
3
4
RB
He-
35S
5S
b)
1µm
1µm
3
Major 35S
CC chromosome
RB
GB
GB
B
1
2
He-
SAT-1
4
B
35S
Eu-
CC4
5
B
GB
B
3
4
He-
He-
SAT-2
6
B
Eu-
Eu-
c)
1µm
2µm
1µm
7
35S
Slide 6: Chestnut rDNA, Islam-Faridi et al. 2024

## Slide 7
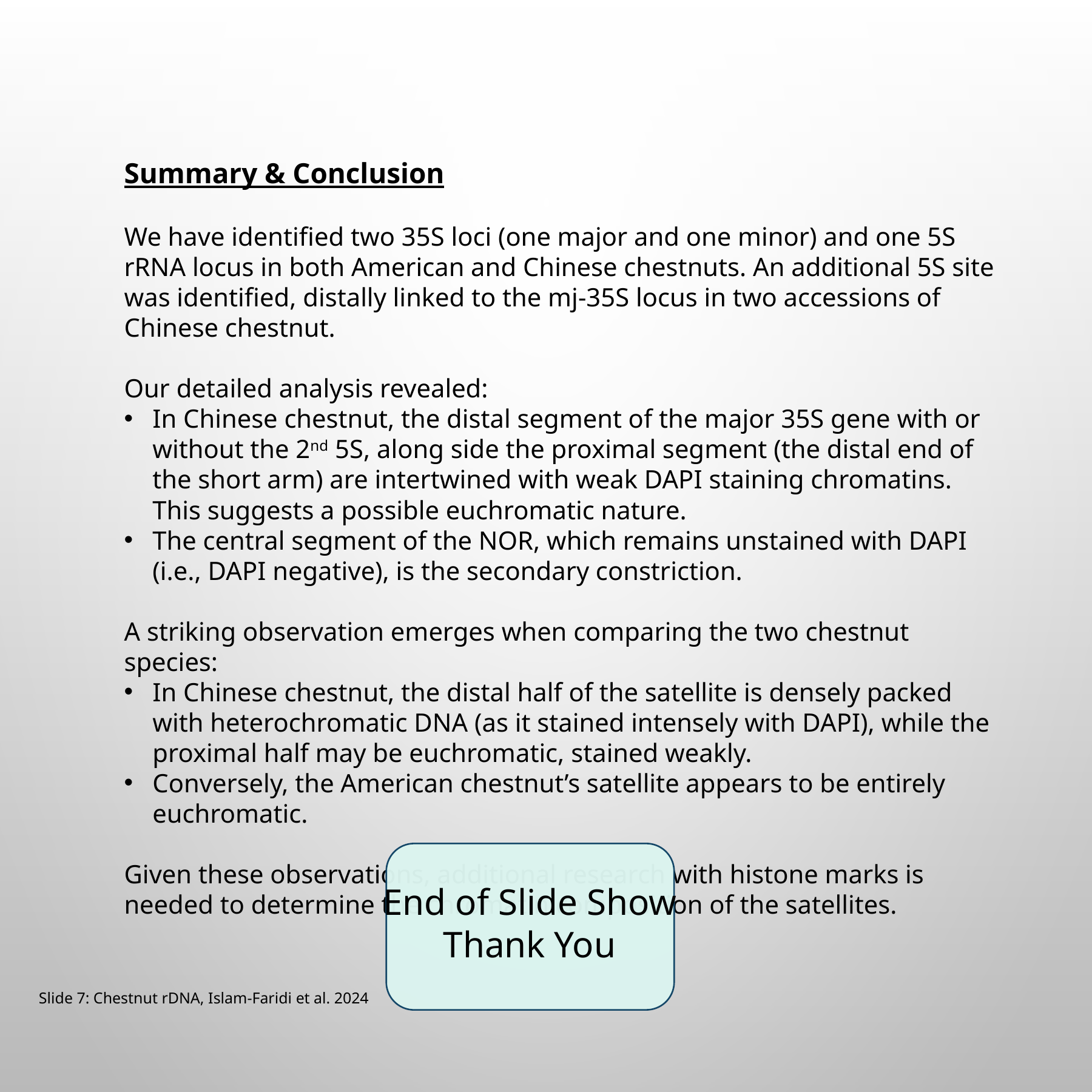

Summary & Conclusion
We have identified two 35S loci (one major and one minor) and one 5S rRNA locus in both American and Chinese chestnuts. An additional 5S site was identified, distally linked to the mj-35S locus in two accessions of Chinese chestnut.
Our detailed analysis revealed:
In Chinese chestnut, the distal segment of the major 35S gene with or without the 2nd 5S, along side the proximal segment (the distal end of the short arm) are intertwined with weak DAPI staining chromatins. This suggests a possible euchromatic nature.
The central segment of the NOR, which remains unstained with DAPI (i.e., DAPI negative), is the secondary constriction.
A striking observation emerges when comparing the two chestnut species:
In Chinese chestnut, the distal half of the satellite is densely packed with heterochromatic DNA (as it stained intensely with DAPI), while the proximal half may be euchromatic, stained weakly.
Conversely, the American chestnut’s satellite appears to be entirely euchromatic.
Given these observations, additional research with histone marks is needed to determine the chromatin composition of the satellites.
End of Slide Show
Thank You
Slide 7: Chestnut rDNA, Islam-Faridi et al. 2024
