# Supplementary material for: Cyto-molecular characterization of rDNA and chromatin composition in the NOR-associated satellite in Chestnut (Castanea spp.)
Source: Sci Rep. 2024 Jan 15;14:980. doi: 10.1038/s41598-023-45879-6 (PMC10789788; doi:10.1038/s41598-023-45879-6)
Supplement: Supplementary file 3 — Supplementary Information 3. [file 41598_2023_45879_MOESM3_ESM.docx]

**Supplementary information – 3**

Slide descriptions/narratives

Cyto-molecular characterization of rDNA and chromatin composition in the NOR-associated satellite in Chestnut (*Castanea* spp.)

Nurul Islam-Faridi^1^, George L Hodnett^2^, Tetyana Zhebentyayeva^3,4^, Laura L Georgi^5^, Paul H Sisco^6^, Frederick V Hebard^5^, C Dana Nelson^7,8^

^1^ Forest Tree Molecular Cytogenetics Laboratory, Southern Institute of Forest Genetics, USDA Forest Service, Southern Research Station, Texas A&M University, College Station, TX 77843 USA.

^2^ Department of Soil & Crop Sciences, Texas A&M University, College Station, TX 77843 USA

^3^ The Schatz Center for Tree Molecular Genetics, Department of Ecosystem Science and Management, The Pennsylvania State University, University Park, PA 16802 USA

^4^ Department of Forestry and Natural Resources, University of Kentucky, Lexington, KY 40546, USA

^5^ Meadowview Research Farms, The American Chestnut Foundation, 29010 Hawthorne Drive, Meadowview, VA 24361 USA.

^6^ The American Chestnut Foundation, 50 North Merrimon Ave., Suite 115, Asheville, NC 28804 USA.

^7^ USDA Forest Service, Southern Research Station, Forest Health Research and Education Center, Lexington, KY, USA 40546

^8^ USDA Forest Service, Southern Institute of Forest Genetics, Harrison Experimental Forest, 23332 Success Road, Saucier, MS 39574 USA.

**Corresponding author:** Nurul Islam-Faridi

E-mail: m.n.faridi@udsa.gov (or nfaridi@tamu.edu); Phone: 979-862-3908

**Animated & automated PPT Presentation**

**Slide 1: Title page**

**Slide descriptions/narratives:** In this animated and automated PowerPoint presentation, we summarize our key findings about the organization and characterization of ribosomal genes, as well as the chromatin makeup of the NOR-associated satellites in both American and Chinese chestnuts. Our research provides new insights into key aspects of cytogenomics, focusing on the NOR and satellite regions, and advances our knowledge of these features in the studied species.

*Click to open the PPT presentation. Then click the left button on your mouse to start the animation (or, press the right or down arrow on your computer keyboard) and wait until the animation stops by itself* ***(~60 sec).***

**Slide 2: NOR – Satellite – Major 35S location.**

The major 35S rDNA, associated with nucleolus, resides in the Nucleolus Organizing Region (NOR), also known as the Secondary Constriction.

Satellites (SATs), small chromosomal bodies, are located beyond the secondary constriction, their length varies across species [1].

Abbreviations

NOR = nucleolus organizing region, S-arm = short arm, L-arm = long arm

The mj-35S site

- is typically sub-terminal (i.e., located near a chromosome's end)

e.g., American accession of Chinese chestnut (we studied four accessions)

- occasionally terminal (located at the chromosome's end)

e.g., American chestnut

- rarely pericentromeric (i.e., close to a centromere)
- e.g., a European accession of Chinese chestnut [3].

“We have proposed six hypotheses regarding the structural rearrangement in chestnuts. For details, see Supplementary Information 1 and Supplementary Fig. S7)”.

Guerra [4] examined 105 plant species and found:

- 91 (~87%) had a sub-terminal 35S site with an associated satellite.
- 14 (~13%) had a terminal 35S site without any satellites.

Typically, NOR-associated SATs are thought to be densely packed with heterochromatin [2]. However, our observations in the Chinese chestnut hint at a departure from this norm. We observed that the proximal region of the satellite is less condensed and may be euchromatic (integrated with the 35S gene, w/ or w/o the 2^nd^ 5S) while distally heterochromatic. We will discuss this observation in greater detail in the subsequent slides.

**Note:** To restart the animation, right-click with your mouse and select "previous", and then left click. The animation will automatically begin.

*Click left on your mouse to start the animation (or, hit the right or down arrow on your computer keyboard), and wait until the animation stops by itself* ***(~130 sec).***

**Slide 3: Distribution and Organization of the rDNA Loci in American (AC) and Chinese (CC2) Chestnuts.**

Our focus here is the distribution of rDNA (35S and 5S) in both American Chestnut (AC) and Chinese Chestnut (CC), particularly the major 35S site. The AC spread is in metaphase, while the CC accession 2 (CC2) is in pro-metaphase. More details will follow in subsequent slides.

Here is a list of abbreviations used in this presentation: RGB = image taken under Red-Green-Blue filters, RB = image taken under Red & Blue filters, B = Blue filter, mj-35S = major 35S rDNA signal, mn-35S = minor 35S rDNA signal, 5S = 5S rDNA signal, mo-ch = mother chromosome, SAT = satellite, NOR = nucleolus organizing region.

1. The image in “a”, taken with RGB filters, shows a highly condensed metaphase cell of AC. It features one pair each of major and minor 35S signals (green) and a pair of 5S signals (red).

The major 35S site is located terminally. Interestingly, we found a small satellite at the terminal end, possibly euchromatic that stained less intensely (weakly) with DAPI (see also Fig. 1; Supplementary Figs. S2 and S3). This was confirmed through image processing and Telo-FISH, despite no prior reports of satellites with terminal 35S sites. Using Telo-FISH, we ascertained the structural integrity of the chromosome.

1. Same cell as in “a” under Blue filter shows enlarged images of the mj-35S bearing chromosomes with SATs indicated by arrows.
2. Image “c” under RGB filters is from CC2, in pro-metaphase, shows 35S (red) and 5S (green) signals. It has the same number of 35S and 5S sites as observed in AC. The satellites, showing 35S signals appear to be detached from their respective mother chromosomes (mo-ch), are encircled in the middle.
3. Same cell as in “c” under a Blue filter shows an enlarged pair of satellites in the upper-middle box, each encircled by oval-shaped dotted line.

*Click left on your mouse to start the next animation (or, hit the right or down arrow on your computer keyboard), and wait until the animation stops by itself* ***(~70 sec)*.**

1. Image “d” displays an enlarged view of the chromosome pair containing the mj-35S rDNA, including the SATs (compare this image with the one in the rectangular box in “c”).

To enhance the visibility of the path of the NOR (i.e. the 35S signal), we further processed the image to adjust the intensity of the red fluorochrome. Additionally, we drew two dotted lines with arrowheads along the trajectory of the 35S signals to illustrate the connection between the respective satellite and its mother chromosome (mo-ch). The image clearly shows that the mj-35S gene originates from the end of the short arm, passes through the secondary constriction, and continues into the proximal region of the satellite.

The SAT pair was enlarged and shown in the rectangular box (middle-left). To enhance the contrast, we further processed the image as well. Two dotted lines were drawn over the path of the red signals that represent the individual chromatids.

*Click left on your mouse to start the next animation (or, hit the right or down arrow on your computer keyboard) and wait until the animation stops by itself* ***(~90 sec)*.**

**Slide 4. Chromatin Composition in the Satellite of Chinese Chestnut Acc. 2 (CC2).**

1. The image under Green & Blue filters presents the mid-prophase chromosome spread of CC2, showing scattered green 35S signals. A pair of satellites highlighted in the upper-middle box.

The satellites of Chinese chestnut are noticeably larger than those of the American chestnut and they have different levels of DAPI intensity. To explore this further, we examined additional CC cells. Interestingly, we found that they all exhibited the same structural attributes: (namely) a large pair of satellites with distinct DAPI intensities. This mid-prophase cell has a chromosome count of 26 (i.e., 24 chromosomes plus 2 detached satellites). The DAPI staining showed distinct intensities: the proximal half of the satellite appeared pale/weak blue under Blue/UV filter, suggesting it may be euchromatic, while the bright-fluorescing distal half is likely heterochromatic. Enlarged image of the satellite pair is shown in panels a1, a2 and a3 for reference.

Note: We routinely capture various stages of cell division with well-distributed FISH signals for further analysis. We recommend fellow researchers in Cytogenomics consider adopting this approach.

a1) The satellite pair shows scattered 35S signals (green) in the proximal region, which stained weakly with DAPI.

a2) DAPI image: The distal, heterochromatic regions of the satellites are encircled with white dotted lines.

a3) The same image as in “a2”: The proximal regions of the satellites stained weakly with DAPI are indicated with braces and highlight possible euchromatic nature.

*Click left on your mouse to start the next animation (or, hit the right or down arrow on your computer keyboard) and wait until the animation stops by itself* ***(~60 sec)*.**

**Slide 5: GISH of Chinese chestnut (CC) and American chestnut (AC) F_1_ Hybrid. Comparative Analysis of NOR, 35S Site and Satellites in American and Chinese Chestnuts.**

1. This image shows an early-metaphase spread from a CC x AC hybrid under Red-Green-Blue filters, where the NOR of both AC and CC are remarkably intact. This interspecies hybrid provides a unique opportunity to examine the compositional differences between the parental satellites under the same biological and cellular conditions. Using GISH combined with 35S rDNA FISH probes, we confirmed that this cell was from a CC x AC hybrid. Attempts to differentiate species' chromosomes using CC DNA as probe and AC as blocking DNA were unsuccessful, suggesting a close genetic relatedness. Despite this, the distinct differences between NORs and satellites confirmed that it is a hybrid.

Additional Abbreviations

CC Sat Ch = diagrammatic illustration of CC satellite chromosome

AC Sat Ch = diagrammatic illustration of AC satellite chromosome

SA = short arm, Cen = centromere, LA = long arm

He- = heterochromatic region, Eu- = euchromatic region, SC = secondary constriction

In AC, the 35S site resides terminally and the signal completely covers the satellite, which is very small. A few yellow spots of signals (blended effect of red and green) are visible at the end of the 35S signal indicate the presence of chromosomal DNA that may be from the small satellite.

In the following sections, through a series of image analyses, we unveil the details of the structural features and chromatin compositions of the NOR and its associated satellites of Chinese and American chestnuts. Pay a closer look at the processed images as they highlight the unique genetic architectures of these regions in these tree species.

An enlarged image of the CC satellite (top left) processed further, reveals the extent of 35S gene integration into the satellite – that confined to its proximal region. The border of the 35S signal, marked by a dotted line, separates the genic region of the satellite (the proximal half) from the bright green fluorescing heterochromatic distal half. In GISH, the signal intensity appears exceptionally distinct compared to the rest of the genome. This region may be species and/or chromosome specific. Additional FISH using clones from this region should support this notion.

*Click left on your mouse to start the next animation (or, hit the right or down arrow on your computer keyboard) and wait until the animation stops by itself* ***(~100 sec)*.**

1. An enlarged image of CC NOR including the satellite (under Red & Blue filters) demonstrates the path of the major 35S gene. It originates at the end of the short arm, proceeds through the secondary constriction, and then into the proximal section of the satellite.
2. Same image as in “b” under Blue filter shows the DAPI stained NOR, including the satellite, divided into four sections. Sections 2, 3, and 4, collectively represent the NOR, and they contain the major 35S gene.

Sections 1 & 2: The entire Satellite shows differential DAPI staining. The proximal half (box 2), less intensely stained, may be euchromatic, while the distal half is heavily stained is likely heterochromatic (compare with the image in ‘b’).

Section 3: Despite being generally known as the "Nucleolus Organizing Region", this is more likely the secondary constriction (SC), and it appears DAPI negative. Three consecutive A-T base pairs are required to bind with DAPI [5], suggesting the absence of such arrangements in this region.

The major 35S sites reportedly do not stain with DAPI (i.e., DAPI negative), but stained brightly with CMA3, suggesting that they are rich in GCs [2, 6]. In fact, it was the secondary constriction rather than the entire 35S site. It is worth noting that the ribosomal genes are high in CGs and have been reported to have CMA3 +ve. Much of the plant FISH data was derived from condensed metaphases, so the structural details were missing (see discussion of the article). In contrast, our findings indicate that the distal (section 2) and proximal (section 4) sections of the mj-35S site are intertwined with chromatin that shows weak DAPI, suggesting that these regions may be euchromatic.

Once again, it is important to note that if FISH images are of good quality and processed appropriately, they can reveal valuable details about the structure and organization of chromosomes.

*Click left on your mouse to start the next animation (or, hit the right or down arrow on your computer keyboard) and wait until the animation stops by itself* ***(~100 sec)*.**

1. An enlarged image of AC NOR under Red & Blue filters, it closely resembles CC. The 35S gene is observed to begin at the end of the short arm, then extend through the secondary constriction, and shrouding a tiny satellite.
2. The same image as in “d” under a Blue filter, shows that the distal two-thirds of the NOR appears DAPI negative. This specific region corresponds to the secondary constriction (SC).

Structural Diagram of the American Chestnut’s 35S rDNA bearing chromosome shown in upper-right in the image “a”.

And for the Chinese Chestnut’s Chromosome shown in lower-left.

Abbreviations used here:

SAT = Satellite, NOR = Nucleolus Organizing Region, SA = short arm, Cen = Centromere, LA = long arm

He- = heterochromatic region, Eu- = euchromatic region

Dotted arrows in the middle of image 'a' point to the diagrams of the short arms of the mj-35S rDNA bearing chromosomes of both Chinese chestnut and American chestnut.

In conclusion, based on our GISH, FISH & DAPI images, the proximal region of the CC satellite is likely to be euchromatic, while the distal half is highly heterochromatic. On the contrary, the American satellite might be euchromatic. Future investigations involving immunostaining with histone marks, should provide more insights into the satellite's chromatin composition.

*Click left on your mouse to start the next animation (or, hit the right or down arrow on your computer keyboard) and wait until the animation stops by itself* ***(~80 sec)*.**

**Slide 6: Chromatin Composition in the Satellite of Chinese Chestnut [Acc. 3 (CC3) and Acc. 4 (CC4)] in Early Phases of Cell-cycle.**

The composition of the DNA in the NOR-associated satellite can be determined during the early phases of the cell cycle, such as interphase and early- to mid-prophase cells, where chromatin condensation varies. In plants the NOR is the last to condense [2]. The FISH images in this animated PowerPoint presentation are from a mid-prophase satellite of CC3 and interphase nuclei of CC3 and CC4. Based on their physical length, we observed two different types of satellites, namely SAT-1 and SAT-2, in CC3 and CC4. The SAT-2 in CC3 is relatively small, while in CC4 the SAT-2 is slightly smaller than SAT-1. For more details, please see the manuscript and supplementary Figure S1B. In this demonstration we will focus on SAT-1 of CC3 and both SAT-1 and SAT-2 of CC4.

Additional abbreviations

SAT-1 = Satellite-1, SAT-2 = Satellite-2

Top left: This diagram illustrates the satellite and nucleolus organizing region (NOR) of the major 35S rDNA-bearing chromosome in Chinese chestnut.

1. This image shows a mid-prophase satellite-1 (SAT-1) of CC3 with the major 35S signal (green) and a faintly visible the 2nd 5S (pinkish-red, which is nearly overshadowed by the green signal). Through appropriately processing FISH images, we can uncover the structural and organizational composition of a genome. For additional information, the image was processed further and presented in panels a2 to a7. These panels provide a deeper understanding of the genomic features observed in the image.

a1) Image under RGB filters: The arrow points at the major 35S signal (green), which occupies the proximal half of the satellite. The distal half stained intensely with DAPI.

a2) Image also under RGB filters: Further image processing (dimmed the green signal), revealing the 2nd 5S gene integrated distally with the major 35S rRNA gene. This organizational complexity can be demonstrated by extended DNA fiber FISH [7] and/or by molecular methods [8]. The white dotted line marks the separation point between the proximal and the distal halves of the satellite.

a3) Image under Red & Blue filters: Image was processed further to eliminate the green signal and enhanced the pinkish-red signals. The 5S signals, one on each chromatid, appeared distinctly visible (dotted oval circle) near the distal peripheral border of the proximal region of the SAT.

a4) Image under Red & Blue filters: Image processed further to dim the pinkish-red signal, two bent arrows show the demarcation boundary between the intensely stained distal region and the weakly stained proximal region of the satellite.

*Click left on your mouse to start the next animation (or, hit the right or down arrow on your computer keyboard) and wait until the animation stops by itself* ***(~40 sec)*.**

a5) Image under Blue filter: The DAPI intensity clearly distinguishes the pale or weakly stained proximal half, may be euchromatic, from the densely stained blue distal half, which is highly heterochromatic.

a6) Same image as in “a5”: The heterochromatic region (He-) of the satellite encircled by white dotted line.

a7) Same image as in “a5”: Both euchromatic (Eu-) and heterochromatic (He-) regions are shown within white dotted enclosures.

*Click left on your mouse to start the next animation (or, hit the right or down arrow on your computer keyboard) and wait until the animation stops by itself* ***(~85 sec)*.**

b) An early interphase nucleus of CC3 showing scattered green signals of the 35S gene and red signals of the 5S gene. A large round condensed heterochromatic DNA body of SAT-1 along with a 5S signal (red, the 2nd 5S site) and scattered 35S signals (green) shown in white dotted box. Highly heterochromatic DNA body reported to remain condensed throughout the cell cycle except during replication in late S-phase [9]. Enlarged images of the same under different filters shown in panels b1 (RGB), b2 (GB), b3 (RB) and b4 (B).

b1) Image under RGB filters: Scattered 35S signals (green) are seen around the condensed heterochromatic body. The 2nd 5S signal (red arrow) along with a pale yellowish signal (a blended effect of red and green) are observed at the periphery of the 2^nd^ 5S signal. In addition, faint green scattered signals were observed between the 5S red signal and the peripheral region of the heterochromatic body. This demonstrates the connection between the condensed heterochromatic body and the decondensed euchromatic DNA (which integrated with the rRNA genes) of the satellite.

b2) Image under Green & Blue filters: Dispersed 35S signals (green) are visible around the heterochromatic body of the satellite.

b3) Image under Red & Blue filters: Two bent arrows clearly show the separation of the red signal (the 2nd 5S site) from the condensed heterochromatic body of the satellite. Thus, this suggests that the 2nd 5S site does not reside within the heterochromatic region of the satellite.

b4) Image under Blue filter: The heterochromatic body (He-) of the satellite is encircled with white dotted line.

*Click left on your mouse to start the next animation (or, hit the right or down arrow on your computer keyboard) and wait until the animation stops by itself* ***(~110 sec)*.**

c) The image represents a late interphase nucleus of CC4 under Green & Blue filters: Two major 35S rDNA signals (green) along with individual condensed heterochromatic DNA bodies (stained densely with DAPI) are shown in white dotted boxes. These heterochromatic DNA bodies are part of the SAT-1 and SAT-2. The SAT-1 is relatively larger than the SAT-2 (for additional details, see the discussion in the main article and Supplementary Fig. S1B). Enlarged images of SAT-1 are shown in panels c1 and c2, while those of SAT-2 displayed in panels c3 and c4, all aiming to provide further insight into the chromatin composition and the precise location of 35S site within the satellites.

c1) Enlarged image of the SAT-1 under Green & Blue filters: The major 35S signal (green, encircled by an oval shaped green dotted line) partially overlaps with the section of the heterochromatic body of the satellite. This is a two-dimensional image and is in a partial polar-side view orientation.

c2) DAPI image, same as in “c1” under Blue filter: The heterochromatic (He-) and euchromatic (Eu-) regions are shown in white dotted circle and brace, respectively.

c3) Enlarged image of the SAT-2 under Green & Blue filters, captured in a side view orientation: The major 35S signal (green, encircled by oval shaped dotted green line) is observed explicitly outside of the large round heterochromatic body of the satellite. This image confirms that the 35S gene integrated with the weakly stained DNA -- the proximal half of the satellite.

c4) A DAPI image (under Blue filter) of the same as in “c3”: The heterochromatic (He-) and euchromatic (Eu-) regions are shown in white dotted circle and brace, respectively.

From these early phases of the cell-cycle study, we conclude that the distal portion of the mj-35S gene with or without the 5S gene, exclusively integrates with the satellite’s weakly DAPI stained chromatin, which may be euchromatic in nature.

*Click left on your mouse (or, hit the right or down arrow on your computer keyboard) to view the Summary and Conclusion and wait until the animation stops by itself* ***(~65 sec)*.**

**Slide 7: Summary & Conclusion**

We have identified two 35S loci (one major and one minor) and one 5S rRNA locus in both American and Chinese chestnuts. An additional 5S locus was identified, distally linked to the mj-35S locus in two accessions of Chinese chestnut.

Our in-depth analysis revealed:

- In Chinese chestnut, the distal segment of the major 35S gene (with or without the 2^nd^ 5S), alongside the proximal segment (i.e., the distal end of the short arm) are intertwined with the weak DAPI staining chromatin. This suggests a possible euchromatic nature.
- The central segment of the NOR, which remains unstained with DAPI (i.e., DAPI negative), is the secondary constriction.

A striking observation emerges when comparing the two chestnut species:

- The Chinese chestnut’s distal half is densely packed with heterochromatic DNA as it stained intensely with DAPI, while the proximal half may be euchromatic, stained weakly.
- The American chestnut’s satellite, however, appears to be entirely euchromatic.

Given these observations, additional research using histone marks is needed to determine the chromatin composition of the satellites.

**References**

1. Battaglia, E. Chromosome morphology and terminology. *Caryologia* **8(1),** 179-187; 10.1080/00087114.1955.10797556 (1955).
2. Zoldos, V. et al. Molecular-cytogenetic studies of ribosomal genes and heterochromatin reveal conserved genome organization among 11 *Quercus* species. *Theor. Appl. Genet.* **99,** 969-977; 10.1007/s001220051404 (1999).
3. Ribeiro, T., Loureiro, J., Santos, C. & Morais-Cecílio, L. Evolution of rDNA FISH patterns in the Fagaceae. *Tree Genetics & Genomes* **7,**1113-1122; 10.1007/s11295-011-0399-x (2011).
4. Guerra, M. Patterns of heterochromatin distribution in plant chromosomes. Genet. Mol. Biol. **23(4)**, 1029-1041. 10.1590/S1415-47572000000400049 (2000).
5. Kapuściński,J. & Szer, W. Interactions of 4, 6-diamidine-2-phenylindole with synthetic polynucleotides. Nucleic Acids Res. **6(11),** 3519-3534; 10.1093/nar/6.11.3519 (1979).
6. Hamon, P. et al. Physical mapping of rDNA and heterochromatin in chromosomes of 16 *Coffea* species: A revised view of species differentiation. *Chromosome Res*. **17,** 291-304; 10.1007/s10577-009-9033-2 (2009).
7. Zoldos, V. *et al.* Epigenetic differentiation of natural populations of *Lilium* *bosniacum* associated with contrasting habitat conditions. *Genome Biol. Evol.* **10(1),** 291–303; 10.1093/gbe/evy010 (2018).
8. Garcia, S. *et al.* Linkage of 35S and 5S rRNA genes in *Artemisia* (family Asteraceae): first evidence from angiosperms. *Chromosoma* **118,** 85–97; 10.1007/s00412-008-0179-z (2009).
9. Estandarte, A. K., Botchway, S., Lynch, C., Yusuf, M. & Robinson, I. The use of DAPI fluorescence lifetime imaging for investigating chromatin condensation in human chromosomes. *Scientific Reports* **6,** 31417; 10.1038/srep31417 (2016).
